# Solvent-activated 3D-printed electrodes and their electroanalytical potential

**DOI:** 10.1038/s41598-023-49599-9

**Published:** 2023-12-20

**Authors:** Karolina Kwaczyński, Olga Szymaniec, Diana M. Bobrowska, Lukasz Poltorak

**Affiliations:** 1 Department of Inorganic and Analytical Chemistry, Faculty of Chemistry, University of Lodz, Tamka 12, 91-403 Lodz, Poland; 2 Faculty of Chemistry, University of Bialystok, Ciolkowskiego 1K, 15-245 Bialystok, Poland

**Keywords:** Chemistry, Materials science

## Abstract

This work is a comprehensive study describing the optimization of the solvent-activated carbon-based 3D printed electrodes. Three different conductive filaments were used for the preparation of 3D-printed electrodes. Electrodes treatment with organic solvents, electrochemical characterization, and finally electroanalytical application was performed in a dedicated polyamide-based cell also created using 3D printing. We have investigated the effect of the used solvent (acetone, dichloromethane, dichloroethane, acetonitrile, and tetrahydrofuran), time of activation (from immersion up to 3600 s), and the type of commercially available filament (three different options were studied, each being a formulation of a polylactic acid and conductive carbon material). We have obtained and analysed a significant amount of collected data which cover the solvent-activated carbon-based electrodes surface wettability, microscopic insights into the surface topography analysed with scanning electron microscopy and atomic force microscopy, and finally voltammetric evaluation of the obtained carbon electrodes electrochemical response. All data are tabulated, discussed, and compared to finally provide the superior activation procedure. The electroanalytical performance of the chosen electrode is discussed based on the voltammetric detection of ferrocenemethanol.

## Introduction

3D printing is a form of additive manufacturing technology that uses a computer-aided design (CAD) model to create three-dimensional objects. Printing is achieved by depositing successive layers of material, such as plastic, polymer, metal, or ceramic until the entire object is created. The technology is used in a wide range of industries^1^, including healthcare^2,3^, automotive^4^, aerospace^5^, and consumer products^6^. It is also becoming increasingly popular for home use, allowing anyone with access to a 3D printer to create objects with a few clicks of the mouse. The dynamic development of 3D printing has led to more and more applications in various scientific fields, including the analytical chemistry laboratories^7–9^. Attempts have been made to print, e.g. sample preparation^10^, separation^11^ and flow^12,13^ systems, sensors and biosensors^14^, or chemical equipment components^15^. The advantages of 3D printing in chemical analysis laboratories cover (i) fast prototyping, (ii) low-cost fabrication; (iii) design freedom; (iv) rapid adjustment to changing experimental conditions; (v) wide selection of printable materials; (vi) scalability reaching the dimensions of the microfluidic systems. 3D printing can also create complex geometries that are difficult to produce with traditional manufacturing methods^16^. In addition, combining 3D printing with materials such as polymers, ceramics, and metals allows the construction of chemical device components with enhanced chemical and mechanical properties. Furthermore, with the help of 3D printing, parts of the designed analytical devices can be quickly replaced and the system can be modified according to the requirements of an individual experiment. In the future, the use of 3D printing in the chemical analysis laboratory is likely to become even more common. 3D printing is expected to play an important role in the development of new analytical instruments, and will facilitate the rapid development of new analytical techniques and methods^7^.

3D printing can also be employed in electrochemistry to create complex 3D electrode structures that can be useful in a variety of applications, especially electrochemical sensing^17–20^. This technology can also be used to create electrochemical cells^21–24^, which are used to perform demanding potentiostatic or galvanostatic experiments. 3D printing drives ongoing revolution in electrochemical research, as the experimental components can be tailored to specific needs and offer a cost-effective and rapid way to produce all needed electrodes (working electrodes, reference electrodes, counter electrodes, etc.)^25,26^. 3D-printed electrodes (3DP) offer several advantages over traditional options, including greater customization options and design flexibility, and short in-the-lab (or on-the-spot) fabricating times^27^. To be more precise, these advantages mainly include: (i) the ability to easily create very complex 3D-geometries; (ii) very rapid prototyping; (ii) low cost of the infrastructure, materials, and needed software’s; and finally (iii) is very user friendly. Mainly due to these reasons, 3D-based electrodes fabrication gains continuously increasing attention. 3D printing techniques that are used for electrode fabrication rely on various materials and printing procedures. Their choice frequently affects the electrochemical properties of the created electrodes. Typical procedures allowing for electrode printing involve the utilization of technology known as Fuse Deposition Modeling (FDM) in which the blend of thermoplastic and conductive particles is used. The electroanalytical cell components can be also created with Inkjet 3D Printing technology. Here, the conductive inks containing materials like silver nanoparticles are cured after deposition over desired surface. A growing number of sensors printed on conductive materials are now being developed to detect various compounds of medical and forensic interest. Among a number of interesting reports, a few elegant examples may be given. Teekayupak et al*.* reported a portable smartphone integrated 3D printed electrochemical sensor for nonenzymatic determination of creatine with a LOD equal to 37.3 μM, which is sufficient to detect creatinine in human urine samples^28^. Another report proposes to use modified 3D-printed electrodes for non-enzymatic detection of tyrosine at 0.25 μM level in human urine samples^29^. Human sweat samples were also analysed for the simultaneous detection of uric acid (0.28 µM) and zinc (1.53 nM L^−1^) with 3D printed PLA/carbon black electrodes^30^. PLA-based graphene blend printed electrodes were used to analyse atropine in beverages at levels as low as 1 µM^31^. A homemade conductive thermoplastic material was developed from carbon black and PLA, and the resulting prints were used for the determination of catechol and hydroquinone by differential pulse voltammetry, giving LOD values of 0.02 and 0.22 µM, respectively^32^. In all these reports, the printed electrodes were a blend of the conductive carbon-based material and the thermoplastic (usually PLA), and hence, the activation step was an inherent part of the entire development process^33,34^. It aims at increasing the surface area of the electrode by exposing the conductive parts of the printout to the contacting solution. The problem of the conductivity of the materials used for 3D printing is one of the main issues that hinders the direct application of 3D printed electrodes. This challenge arises because the conductivity of the materials directly impacts the performance and functionality of the printed electrodes mainly due to the presence of thermoplastic which acts as the carrier of the conductive material. In the context of 3D printed electrodes, materials with poor electrical conductivity can result in inefficient energy transfer, and reduced electrode performance. Improving the conductivity of 3D printable materials involves various strategies, such as incorporating conductive additives like graphene or metallic nanoparticles into the printing filaments. Additionally, optimizing printing parameters, such as layer thickness and infill density, can also influence the overall conductivity of the printed electrodes. Activating the surface of 3D printed electrodes is a critical step in enhancing their performance and functionality. The conductivity of the material alone is not sufficient; an activated surface is essential to facilitate efficient electron transfer and chemical reactions in various applications. By activating the surface, we create more active sites for electrochemical reactions, which can significantly boost the sensitivity and responsiveness of sensors. 3D-printed electrodes can be activated by a variety of methods. Depending on the material used for printing, the activation method may vary. For example, 3D-printed carbon-based electrodes can be activated by mechanical polishing^35,36^, electrochemically (by applying an anodic potential to oxidize and then a cathodic potential to reduce the surface)^37,38^, and solvent activation^34^ among many others. The latter methodology involves the use of a solvent that partially dissolves the thermoplastic material, resulting in the exposure of the conductive components. This methodology is especially attractive since different solvent will act on the thermoplastic differently, introducing a control factor allowing to custom surface properties adjustment. To the based on our knowledge, the effects of polar aprotic solvents (dimethylformamide and acetone) and polar protic solvents (ethanol, methanol, and water) action on the surface activation of 3DP electrodes (PLA-graphene blend; Black Magic) have been studied. It was proven that polar aprotic solvents have better activation capabilities than polar protic solvents^34^.

In the present work, we have used three different conductive, carbon-based filaments for the working electrodes development. Before final use, all electrodes were subjected to the action of five solvents—acetone, dichloromethane, dichloroethane, acetonitrile, and tetrahydrofuran. The effect of the solvent on the 3D-printed electrode surface was examined in function of time. The changes in the electrode surface properties were studied with contact angle measurements, scanning electron microscopy, atomic force microscopy, and finally cyclic voltammetry. Obtained data are tabulated, analyzed and the optimized procedures for the activation of the 3D-printed electrodes are proposed. The electroanalytical potential of the selected electrodes was tested in the presence of ferrocenemethanol using cyclic voltammetry and differential pulse voltammetry. Obtained results indicate, that with the choice of proper solvent-based activation procedure, we can control the 3DP electrode surface roughness and its wettability, which are crucial parameters affecting the output of the electroanalytical experiment.

## Methods and materials

### Chemicals

Tetrahydrofuran (THF, 99.5%), dichloromethane (DCM, 99.8%), and acetone (AC, 99.5%) were purchased from POCh (Poland). Dichloroethane (DCE, > 99%) and acetonitrile (ACN, 99%) were obtained from Sigma Aldrich and Honeywell, respectively. All solvents were used as received. Sodium nitrite was purchased from Chempur (Poland), and ferrocenemethanol (FcMeOH, 97%) was obtained from Sigma Aldrich. All aqueous solutions were prepared using demineralized water (Hydrolab system, 0.055 µS cm^−1^, Poland).

### 3D printing fabrication

**Figure 1:**
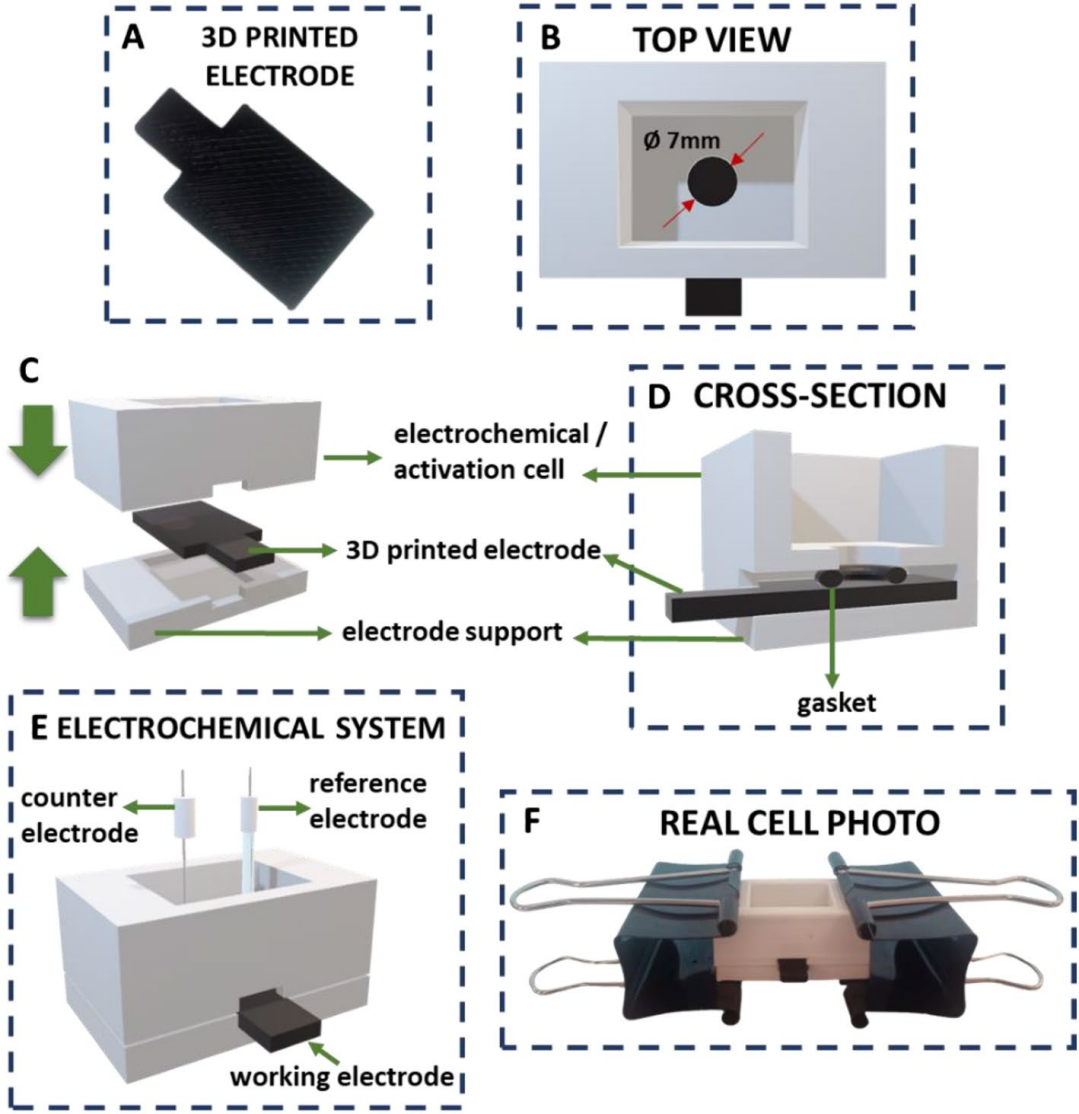
Image of (**A**) 3D printed electrode. Schemes showing (**B**) the top view of the electrochemical cell with the mounted electrode, (**C**) a sequential representation of the electrochemical cell modules sandwiching the electrode, (**D**) a cross-section of the measuring cell indicating the position of the O-ring, and (**E**) the entire electrochemical set-up used during electrode voltammetric characterization. (**F**) Shows the real cell photo.

All designs were created using Tinkercad software, and were further exported as .stl files. The selection of the optimal shape of the 3D printed working electrodes was based on the design of several printouts using a non-conductive neutral polylactic acid filament (PLA, Nebula Filaments, Poland). The final and optimized dimensions of the electrode were fixed to 15 mm–28 mm–2 mm (width–length–height, respectively) (see Fig. 1A,B for details). Conductive filaments: Proto-pasta (ProtoPlant Inc., Canada), Prografen Graphene (Advanced Graphene Product SA, Poland) and Ampere (Print-me, Poland) were used to print the working electrodes in their final shape adjusted to electrochemical, microscopic (SEM and AFM), and contact angle measurements (see Fig. 1A). The conductive component as claimed by the manufacturer was different in each employed filament (Proto-pasta—carbon black, Prografen—graphene flakes, Ampere—carbon nanotubes (CNTs)), suggesting different electrochemical properties. The electrodes printed from the neutral PLA were used during the control studies. All electrodes were printed with Prusa i3 MK3S (Prusa, Czech Republic) 3D printer. The .gcode file for the electrodes was prepared and exported using PrusaSlicer 2.5.2. software with the specified parameters: layer height 0.10 mm, filling density 100%, extruder temperature 220 °C, and table temperature 60 °C. The electrochemical/activation cells used in this work were fabricated using Polyamide 12 (PA12, white, Fiberlogy) filament and Polyvinyl Acetate (PVA, Rosa3D, Poland). The choice of PA12 was dictated by the high chemical resistance of this material, needed during the electrode activation process. AC, DCM, DCE, THF, and ACN used during the activation step did not affect the cell printed using PA12. Neither dissolution nor swelling was observed even after prolonged (a few hours) exposure times. During the cell printing, it was also necessary to introduce the PVA filament, which acted as a sacrificial support for the unsupported PA12 cell parts. After printing, PVA was dissolved using water finalizing the printing process. Simultaneous printing with both filaments was conducted with Creator Pro2 printer (Flashforge, China) equipped with two printing heads, whereas for the slicing (.gcode generation) we used FlashPrint 5 software. The following parameters were applied for the cell printout: layer height 0.18 mm, filling density 15%, 7 contours (assuring cell walls impermeability to organic solvents and aqueous solutions), extruder temperature (PA12) 230 °C, extruder temperature (PVA) 200 °C, and table temperature 100 °C. Since the cell fabrication was based on two filaments printing we have applied a cleaning wall (print sheath) significantly reducing a number of geometrical artifacts in the final printout.

The electrode surface activation and dimensions of the electrode during electrochemical characterization remained constant during the experiments as a single cell was used for both steps (see Fig. 1B–D). The activated and geometrical area of the electrode is assumed to take the shape of the circle printed in the upper module of the electrochemical cell equal to 7 mm—see Fig. 1B. The measuring vessel was split into two separate sections, i.e. the upper part which was the electrochemical/activation cell hosting an activation solvent or the aqueous phase during the measurements (maximal volume of the module was 4 mL), and the bottom part (electrode support) with an electrode cavity. During the processing, the electrode was pressed in between two cell modules with a gasket additionally placed between the electrode and the upper part of the cell (the position of the gasket is depicted in Fig. 1C,D). Tight pressing the electrode in between two modules with two office clips was sufficient to create leak-proof contact between all cell parts. The real photo of the assembled electrochemical cell is shown in Fig. 1F.

### Activation procedure

The solvent activation procedure consisted of contacting the selected solvent with the printed electrode surface for a specified time (dip, 20 s, 40 s, 60 s, 120 s, 300 s, 600 s, 1200 s, 1800 s, and 3600 s). The activation was performed in a 3D printed cell depicted in Fig. 1. For each time, the surface of the electrode exposed to the action of the chosen solvents was fixed and had the shape of a circle 7 mm in diameter. Five different solvents (THF, DCM, DCE, ACN, AC) were used to partially dissolve the non-conductive PLA, exposing the conductive carbon-based particles. After the pre-defined time, the organic solvent was drained from the cell and the resulting electrode surface was thoroughly rinsed with demineralized water, and dried under the stream of compressed air.

### Electrochemical measurements

Electrochemical measurements (cyclic voltammetry (CV), and differential-pulse voltammetry (DPV)) were performed using a potentiostat Emstat^3^ (Palm Instrument B. V., Netherlands) controlled with PSTrace 5.8 software. A 3D printed electrode, silver/silver chloride electrode (Ag/AgCl/3M KCl, Mineral, Krakow, Poland), and a platinum wire (Pt, 99.99%, The Mint of Poland, Warsaw, Poland) were used as the working, the reference and the counter electrodes, respectively. The electrochemical configuration of the employed set-up showing the electrodes and 3D printed cell is schematically depicted in Fig. 1E. Cyclic voltammetry was used to study the electrochemical behavior of FcMeOH at activated and non-activated 3D printed carbon-based electrodes in the potential range of − 0.5 to 0.7 V, and the scan rate of 100 mV s^−1^.

### Contact angle measurements

ThetaFlex optical tensiometer by Biolin Scientific was used during contact angle measurements of the surfaces of 3D printed electrodes before and after solvent activation. Before measurements *ca.* 5 µL drop was dripped onto the surface using a Hamilton syringe 1001 TPLT.

### Scanning electron microscopy and atomic force microscopy

Scanning electron microscopy (SEM), and atomic force microscopy (AFM) were used to study the surface properties of the 3DP electrodes before and after the solvent activation step. SEM images were taken with FEI INSPECT S50 microscope. The accelerating voltage of the electron beam was set to 15 keV while the working distance was set to 10 mm. All samples were attached to the specimen with a carbon tape. Atomic Force Microscopy (5500 AFM, Agilent Technologies) operated in a tapping mode in an air atmosphere. Silicon-sensor with resonance frequency between 45 to 115 kHz (NANOSENSORSTM) served as a cantilever.

## Results and discussion

Three conductive filaments were tested and used to print the designed working electrodes. For all three employed thermoplastic blends, the PLA served as a base polymer. The commercially available products contain different forms of carbon, i.e. carbon black (Proto-pasta), graphene flakes (Prografen), and carbon nanotubes (Ampere). The given statement is based on the manufacturer's declaration. According to the specification, Proto-pasta has a resistance of 2–3 kOhm for a 10 cm length of 1.75 mm filament, whereas, for the other two, the electrical resistance value was not given. It is worth noting that the tested filaments were visually different before and after printing (see Fig. S1). Carbon-based filaments from Proto-pasta and Prografen gave printouts with shiny and smooth surfaces, and did not cause any printing problems. Filament by Ampere, on the other hand, had a different appearance (matte surface) was brittle, and caused printing difficulties with a clogging nozzle being a major issue. Before and during printing, all carbon-based filaments were stored at 50 °C, which improved printing process repeatability. Printed electrodes were subjected to a solvent activation procedure in all five chosen organic solvents, i.e. THF, DCE, DME, ACN, and AC all capable of dissolving PLA^39–41^ and further exposing conductive carbon-based particles to the electrode surface. Changes on the surface of printed electrodes for bare PLA (control) and carbon-based PLA conductive filaments after contact with solvents, were visible to the naked eye already starting from the initial activation times. Figure S2 shows real photos of printouts made out of neutral PLA only (Fig. S2A), Proto-pasta (Fig. S2B), Prografen (Fig. S2C), and Ampere (Fig. S2D), exposed to the action of applied solvent for a specified amount of time. As expected, the solvent PLA dissolution capability varied. The printouts immersed in DCM degraded rapidly. Already after 2 min (Fig. S2B) the electrodes were dislodged forming a suspension (the case of Proto-pasta). For other solvents, the prolonged exposure times reaching 1h, affected the electrodes appearance, as bents, holes, and other defects can be noticed at their surface (Fig. S2A,C,D). The use of DCE had a similar effect on the prints as DCM, but the degradation of the print was not as severe. THF, ACN, and AC dissolved PLA on the surface of the prints, leaving a white residue, associated with dissolved and further re-crystalized PLA. The set of activated electrodes was further characterized using contact angle, SEM, AFM, and voltammetry measurements aiming at a comprehensive understanding of the correlation between the type of the used solvent and electroanalytical properties of the employed 3DP electrodes.

### Wettability characterization

**Figure 2:**
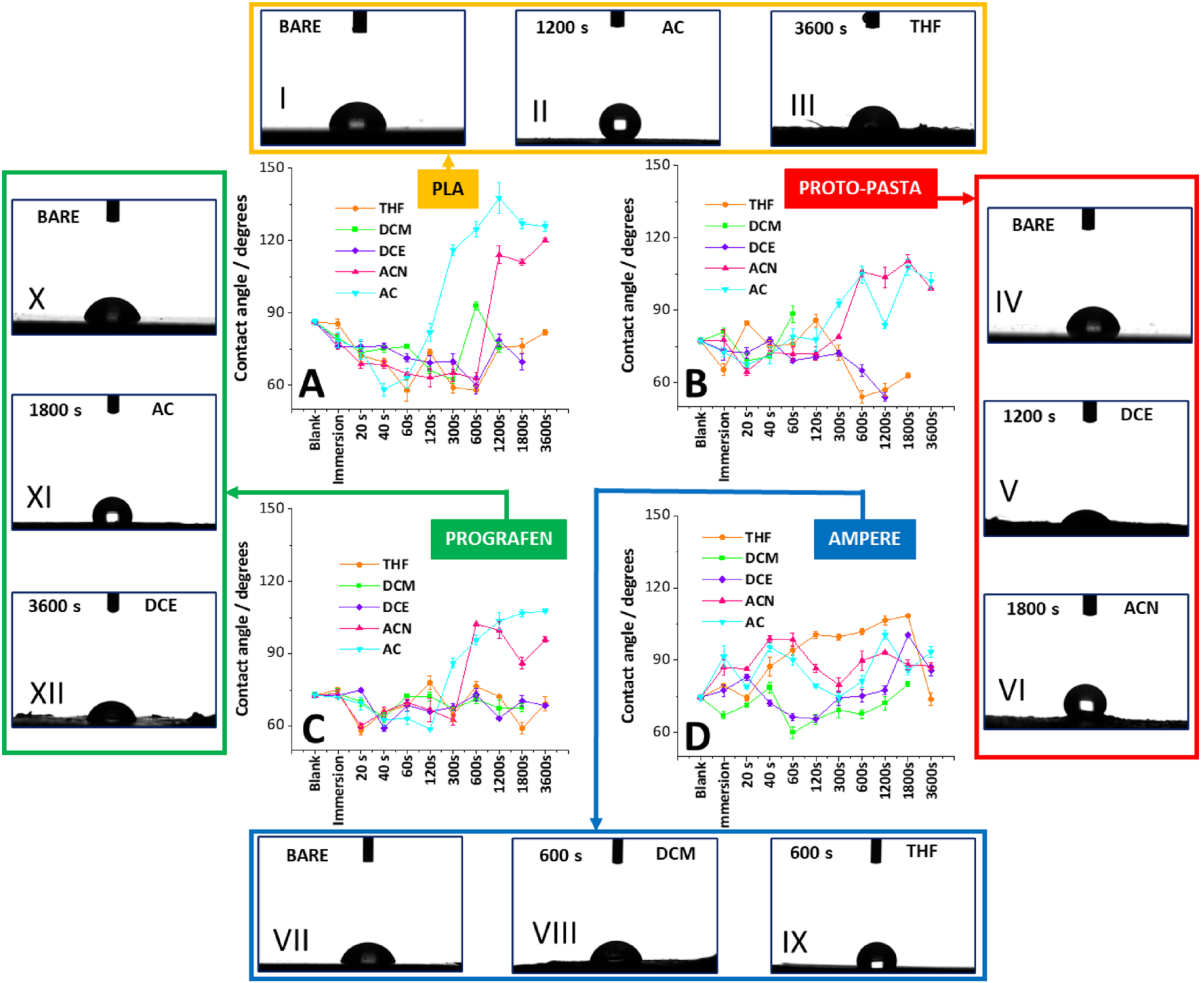
Contact angle measurements plotted in function of the solvent activation time of the 3D printed plates/electrodes using (**A**) bare PLA, (**B**) Proto-pasta, (**C**) Prografen, and (**D**) Ampere. Datapoints correspond to the following solvents: THF (●, orange), DCM (■, light green), DCE (◆, violet), ACN (▲, pink), and AC (▼, cyan). Error bars are the standard deviation calculated from 3 measurements. Numbers I to XII represent images taken for droplets placed over the 3D printed plates/electrodes: I—bare PLA, II—PLA activated in AC for 1200s, III—PLA activated in THF for 3600 s, IV—bare Proto-pasta, V—Proto-pasta activated in DCE for 1200 s, VI—Proto-pasta activated in ACN for 1800 s, VII—bare Ampere, VIII—Ampere activated in DCM for 600 s, IX—Ampere activated in THF for 600 s, X—bare Prografen, XI Prografen activated in AC for 1800 s, and XII—Prografen activated in DCE for 3600 s.

The change in hydrophobicity/hydrophilicity of the surface of the 3DP electrodes was monitored as a function of activation time, type of an employed solvent, and filament used during the fabrication process. In this respect, we monitored the contact angle of the aqueous droplet placed at the electrode surface which was exposed to the action of concerned solvents. First, we have investigated printouts made out of PLA-based filament before and after immersion (2 s), and solvent treatment for the varied amount of time: 20 s, 40 s, 60 s, 120 s, 300 s, 600 s, 1200 s, 1800 s, and 3600 s. All used solvents can dissolve PLA. In general, polymers do not dissolve instantaneously. The dissolution process involves the diffusion of the solvent molecules into a semicrystalline polymeric structure, polymeric chains unfolding, and finally disentanglement. Solvents displaying different solvent power affects the conformational structure adopted by the polymer, either through chemical interactions or via physical processes such as swelling and cracking being a consequence of the non-uniform distribution of solvent molecules diffusing into a polymeric framework^42,43^. The resulting conformational changes can be assessed by measuring the surface hydrophobicity/hydrophilicity of the films^41^. The measured average contact angle values for the electrodes not treated with a solvent were 86° (Fig. 2I), 77° (Fig. 2IV), 73°(Fig. 2X), and 75° (Fig. 2VII) for bare PLA, Proto-pasta, Prografen, and Ampere, respectively. The presence of carbon-based additives in the conductive PLA filaments increased the hydrophilicity of the surface which is consistent with what has been already reported by others^44^. Jaseem et al*.* showed that the hydrophilicity of the PLA-based electrodes increased as the carbon black loading was elevated. It was stated, that the presence of conductive additives affects the promotion and dissipation of static electricity of PLA affecting its interaction with water molecules^45^. The contact angle of pure graphene was found to be 42° ± 3°^46^, whereas for the film made out of CNTs^47^ with an average diameter between 50 and 126 nm the contact angle varied from 54° to 79°. Graphene and CNTs are claimed to be present in the Prografen and Ampere filaments, most probably as additives to other conductive materials such as carbon black or graphite. The exact composition of both materials is not given by the manufacturer (graphene and nanotube loading is unknown), as such the observed drop in the contact angle most probably originates from the combined effect of all conductive constituents present in the filament. We have performed a comprehensive study of the bare PLA and PLA loaded with conductive carbon material surface wettability after exposing printed plates to different organic solvents for a successively increasing time.

A particularly noticeable increase in the surface hydrophobicity of PLA-based printouts surface was observed after treatment with AC (as also reported by others)^48^ and ACN. The contact angle equal to 116° was obtained already after 300 s and increased up to 138° (nearly ultra-hydrophobic surface) for the 1200 s (see Fig. 2-II) when the printed plates were treated with AC. The surface hydrophobicity of the PLA plates treated with ACN significantly increased from 63° up to 114° measured after 600 and 1200 s of the exposure time (see Fig. 2A), and remained at a high level for 1800 s (111°) and 3600 s (120°). The printed plates treatment with three other solvents, this is THF, DCE, and DCM has led to a progressive increase of the printed PLA plates hydrophilicity down to around 60° for the first 600 s. At higher activation times, the contact angle has slightly increased (see the data points for times equal from 1200 to 3600 s). The missing points in the tendencies recorded for DCM and DCE are due to plate dissolution. In the next step, we printed the electrodes using Proto-pasta, Prografen, and Ampere filaments. All 3DP electrodes were subjected to the action of five solvents for the predefined time. The contact angle measured for all activated surfaces reflected similar tendencies as compared with those obtained for bare PLA plates. A few interesting observations were made: (i) for Proto-pasta and Prografen the highest surface hydrophobicity (in the range from 90° to 110°) was obtained when AC and ACN were used as the activation solvents (Fig. 2B,C). These values are noticeably lower than the ones obtained for bare PLA and most probably are due to the presence of carbon-based conducting additives. (ii) DCM displayed the most profound solvent power as already after the first few minutes it was capable of dissolving electrode 3D printed using Proto-pasta (Fig. 2B). Times > 1800 s were needed to dislodge the polymers making the electrodes printed with Prografen and Ampere (Fig. 2C,D). (iii) The electrodes printed using Ampere, treated with THF displayed a progressive increase of the contact angle starting from 75° up to around 108° measured for 1800 s. (iv) For all three electrodes, and especially for the Proto-pasta, the solvent with very high PLA dissolution capabilities—DCE, allowed for the increase of the surface hydrophilicity within the first 600 s. Since carbon-based materials have a higher affinity to aqueous solvents than PLA alone (a surface made out of carbon particles is more hydrophilic than PLA), the obtained data may indicate what is the ratio of two different types of materials that are exposed to the contacting water droplet. As such, we have concluded that the solvent with a high solvation power, DCE, and DCM rapidly entangles the polymeric framework, leaving carbon-based particles exposed at the surface, which in consequence results in the hydrophobicity drop. The solvents such as AC and ACN were found to be significantly weaker than chlorinated alternatives^40,49–51^. As such, the PLA dissolution kinetics and consequent exposure of the carbon-based particles should be significantly slower. As a matter of fact, for these two solvent, the surface hydrophobicity among all studied 3D printed objects (PLA plates and the electrodes) were always significantly higher than the reference value 86° found for bare PLA. This observation indicates that either conformational changes are happening at the electrode surface with the hydrophobic domains being exposed to the contacting activation solvent or the surface architecture is being affected which in consequence affects the printout wettability (existence of pores which act as the air pockets—see SEM images analysis vide infra)^52^.

### Microscopic characterization

#### SEM characterization—Proto-pasta-based electrodes

**Figure 3:**
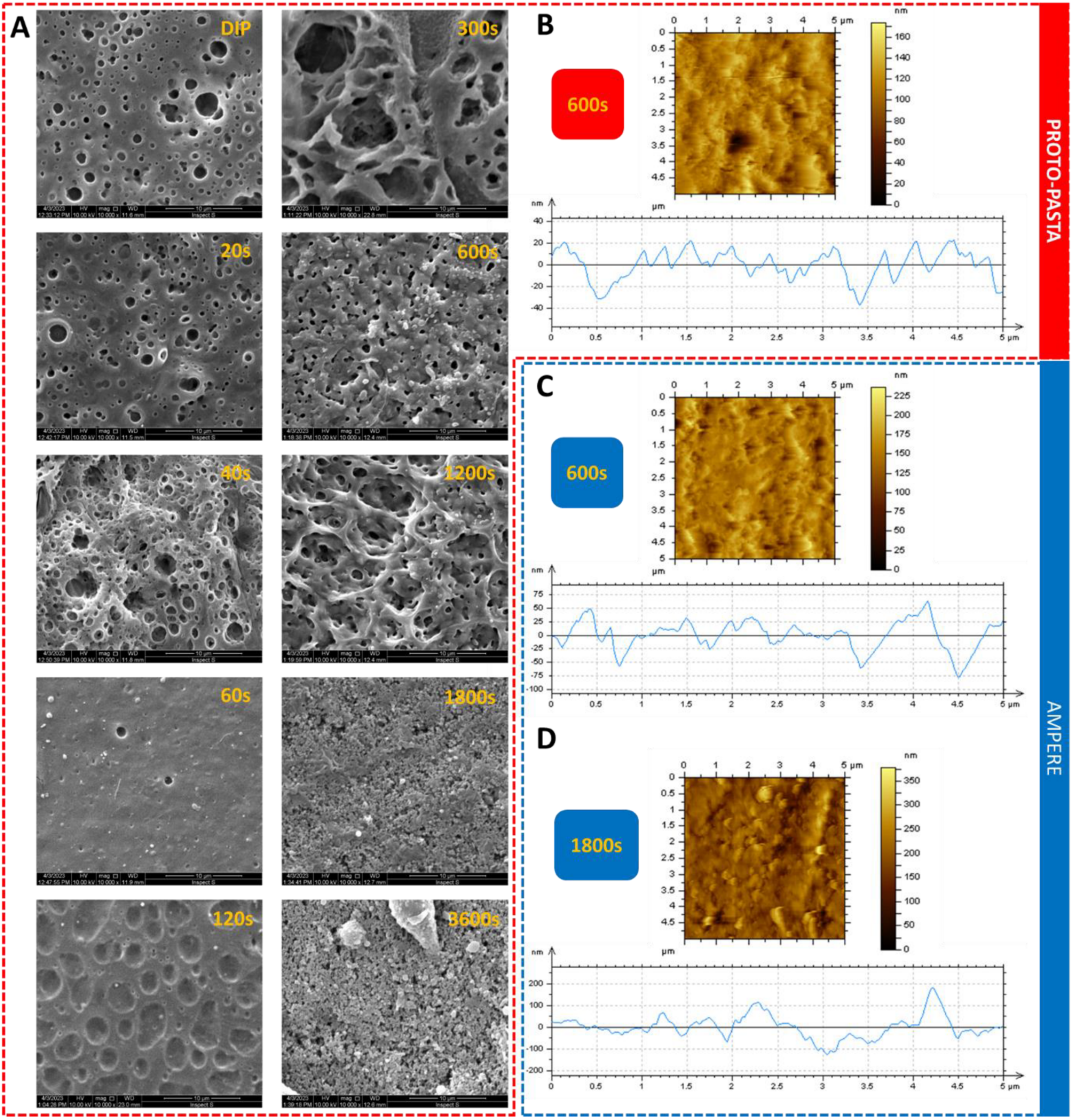
(**A**) SEM images of 3DP electrodes (Proto-pasta) after THF–activation (the solvent activation time is indicated in the right upper corner of the SEM images). Magnification: 10,000×. AFM images and corresponding surface roughness height profiles of 3DP electrodes THF–activated made from (**B**) Proto–pasta after 600 s and Ampere after (**C**) 600 s and (**D**) 1800 s.

Next, we analyzed the surface of the 3D-printed electrodes with scanning electron microscopy and atomic force microscopy after their activation in the 3D-printed cell. The goal was to inspect the resulting surface and correlate obtained data with the contact angle (vide supra) and electrochemical performance (vide infra). Figure 3A shows SEM images taken for 3D printed electrodes made with Proto-pasta after THF-activation at different times of solvent exposure. The PLA-based samples were sputter-coated with gold to prevent a charging effect observed before^53^. No particular differences in the surface texture were observed for the samples subjected to THF immersion and 20 s-long treatment. The longer solvent exposure times resulted in significant dissolution of the thermoplastic, as shown by an increase in the number and size of pits, pores, and holes. The surface of the activated electrodes appears to become increasingly rough, and after 1800 s almost complete removal of the insulating material (PLA matrix) generated a highly porous surface originating from the exposure of the carbon-based particles^54–56^. These results are in line with the conclusions drawn from the surface wettability studies. The longer exposure time of the printouts to THF has led to an increase in the hydrophilic properties of the surface due to hydrophobic domain depletion. The dissolution of the insulator coupled with the exposure of the conductive particles can be further confirmed by increasing peak currents (anodic and cathodic) originating from ferrocenemethanol (see Fig. 5A,B) observed for elongated THF activation times. SEM images were also recorded for samples printed with Proto-pasta (Fig. S3A), Prografen (Fig. S3B), and Ampere (Fig. S3C) after 120 s activation for all employed organic solvents. Significant differences for different filaments treated with the same solvents (see rows from I to V), or different solvents for a single material (see columns from A to C) were observed. Electrodes printed using Proto-pasta activated at 120 s in THF and AC are the roughest (highest number of pits) among all tested solvents which further translates into high electrochemical activity (high electroactive surface area gave high current signals). Here, we have also observed that the water contact angles were significantly higher than after activation with the other solvents (presumably, the porosity contributed to the existence of the air pockets that may decrease the surface wettability).

#### SEM characterization—Ampere-based electrodes

The SEM images shown in Fig. S3 indicate that Ampere electrode surfaces activated with THF, AC, and ACN at 120 s display a number of fine features that were attributed to carbon-based particles. Such features could not be observed for other materials (see e.g. Fig. S3I-A; III-A or III-B) or Ampere-based electrodes activated with DCM (Fig. S3II-C) or DCE (Fig. S3III-C). This is also in line with the electrochemical characterization, as the recorded peak currents were significantly higher for THF, AC, and ACN than when chlorinated solvents were used during activation. Also, the electrodes activated with THF, AC, and ACN gave high values of contact angles indicating elevated hydrophobicity.

### SEM characterization—Prografen-based electrodes

Finally, the case of solvent-activated Prografen is shown in Fig. S3 column B. For the sake of comparison with other 3D printed electrodes, we have focused on 120s activation time. For all used solvents, pitting and holes of various sizes are visible, whereas, in the case of DCE activation, aggregates are additionally present. The latter may originate from the first dissolving and then recrystallizing PLA (Fig S3II-B). In general, the hydrophobic/hydrophilic properties of the activated Prografen electrodes surface did not vary significantly depending on the solvent used, and neither did their electrochemical properties.

#### AFM characterization

AFM images showing the corresponding surface roughness height profiles are shown in Fig. 3B–D. We have found that the THF exposure of Proto-pasta (Fig. 3B) and Ampere (Fig. 3C) leads to the rougher of the latter. Also, the surface roughness analysis has revealed (and further confirmed the inspection of SEM images) that the longer the exposure time (in this case THF) the rougher the surface is (see Fig. 3D). Electrode activation with DCE (activation time—120 s), also induced a rougher surface for Ampere (Fig. S4B) as compared with Proto-pasta (Fig S4A).

### Electrochemical characterization

Activation of the PLA-based conductive filaments involved the partial dissolution of the PLA, so PA12 was used to print the electrochemical cell serving as the activation module and further for the electrochemical 3D printed electrodes characterization. PA12 was selected due to its high chemical resistance to organic solvents (even chlorinated). We did not observe either dissolution or swelling of the 3D printed cell even for prolonged electrode surface activation times in a solvent like DCM or DCE. Utilizing a single cell for activation and electrochemical studies ensured that the electrochemically activated surface area of the 3DP electrode remained constant (7 mm diameter circle) during all measurements. It is worth emphasizing that fabricated cell has been used multiple times, without compromising on their quality. Also, the cell was designed in a way that minimized the volume of solvents that were used during electrode activation (around 1 mL). Initial electrochemical evaluation of the 3D printed electrodes activated surfaces was performed with Fe(CN)_6_^3−/4−^ aqueous solution as a redox probe, but the results were not satisfactory (large peak to peak separation  ≫ 59 mV, significant resistance manifested by titled anodic and cathodic peak current signals, small current values attributed to the anodic and cathodic reactions). FcMeOH was found to be a redox probe giving a measurable electrochemical response already for the non-activated carbon-based PLA electrodes. This behavior can be connected with the relatively high electrode surface hydrophobicity, as such facilizing the interaction between the electrode surface and lipophilic analyte (logP_ow_ for FcMeOH was reported to be 2.1, logP_ow_ for Fe(CN)_6_^3−/4−^ was calculated to be − 4.76 (using platform https://www.molinspiration.com/)^57,58^. As discussed above the surfaces of the solvent-activated electrodes were relatively hydrophobic, and hence the non-polar interactions may facilitate the adsorption of hydrophobic molecules. This results in the molecules being held at the surface via weak van der Waals forces. Therefore, the redox probe with elevated hydrophobicity was expected to give a superior electrochemical response^59^.

#### Effect of solvent activation on the electroanalytical performance of the electrodes

**Figure 4:**
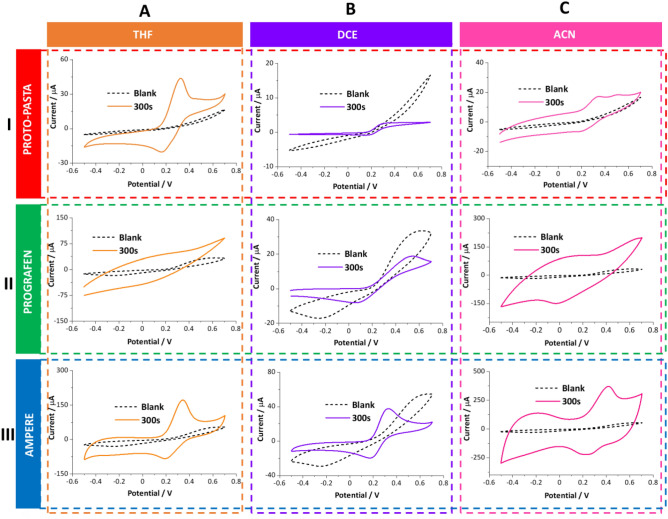
Cyclic voltammograms (CVs) recorded in the aqueous solution of 1 mM FcMeOH in 0.1 M KCl before (non-activated surfaces—black, dashed lines) and after 3D printed electrodes activation with an indicated solvent (solid lines: orange—THF, violet—DCE, pink—ACN). The activation time was set to 300 s. The filament type is indicated in the left panel of the figure. The scan rate was 100 mV s^−1^. The anodic scan was set as the forward polarization.

We started with collecting the CV data for the electrodes printed using all three conductive filaments recorded in the presence of 1 mM FcMeOH. Before activation, CVs were recorded using non-treated electrodes to assess their electrochemical performance (reference curves are indicated as “blank” in Fig. 4). The resulting curves were then considered as a reference for the solvent-activated electrode performance. Activation of the electrode surface was carried out by exposing the surface of printed electrodes to five selected solvents for a specified time (2 s—immersion, 20 s, 40 s, 60 s, 120 s, 300 s, 600 s, 1200 s, 1800 s, and 3600 s). Figure 4 shows the selected curves recorded for electrodes printed with Proto-pasta (row I), Prografen (row II), and Ampere (row III) before and after activation (300 s) in (A) THF, (B) DCE, and (C) ACN. For the data set showing the uniform y-axes further indicating changes of the electroactive surface please refer to Fig. S5 from electronic supporting information. Identical tests were also carried out using DCM (exception, 120 s for Proto-pasta due to PLA dissolution at 300 s) and AC (see Fig. S6). The shape of the current–potential patterns recorded for the electrodes printed using Proto-pasta before activation reflects the behavior of a resistive surface without any clear redox characteristics that could be attributed to FcMeOH oxidation or FcMeOH^+^ reduction (see CVs marked with the black line from Fig. 4AI–AIII). Completely different voltammetric characteristics were obtained when the 3DP Proto-pasta electrodes were activated with all five employed solvents. In each case, the pair of signals with the clearly decreased charge transfer resistance were recorded (see Fig. 4I and Fig. S6). Further analysis of the plotted data and their comparison with the blank reading indicated that (i) the peak-to-peak separation approach around 100 mV for DCM, DCE, ACN, and THF; (ii) AC provided the highest increase in the anodic and cathodic peak currents being around tenfold times higher as compared with other voltammograms but compromised by the higher resistivity of the created electrode.

Figure 4II shows a set of data recorded for the Prografen before and after activation with THF, DCE, and ACN (for the results obtained when DCM and AC were used referred to Fig. S6). We have observed that even non-activated electrodes can be used to follow the FcMeOH oxidation/reduction, although highly resistive voltammograms are recorded as indicated by the exceptionally high peak-to-peak separation of about 0.7 V. 3D-printed electrodes activation with THF (Fig. 4II-A), ACN (Fig. 4II-C), and partially with AC (Fig. S6) was unsuccessful. We have observed substantial separation of the capacitive currents significantly overlaid with the Faradaic currents attributed to FcMeOH redox reactions. High capacitance currents can be attributed to induced surface porosity, as confirmed with SEM analysis. Activation with chlorinated solvents resulted in a significant reduction of the peak-to-peak separation still being four times higher than the expected 59 mV. Also, as one can notice, the anodic and cathodic peak intensities have dropped, which in turn may suggest a smaller electroactive surface area. Nevertheless, it seems that the chlorinated solvents are a preferential choice for the Prografen-based 3DP electrode fabrications among five tested activators. Finally, we evaluated the effect of five organic solvents on the performance of the electrodes printed using the Ampere filaments. The shape of the voltammogram recorded before activation resembled the characteristics that are already described for Prografen. As visualized with the set of data from Fig. 4-III and Fig. S6 activation of these electrodes in any solvent significantly improved the voltammetric features.

Although AC and ACN activation increased the capacitive current which is linked to elaborated surface area (see SEM and AFM from Figs. S3C and S4) and exposed carbon particles^60^ we were still able to detect clear signals attributed to the FcMeOH oxidation/reduction. Nevertheless, these THF, DCM, and DCE gave superior electroanalytical features after electrode surface activation.

#### Effect of activation time

CVs recorded in the presence of a fixed concentration of FcMeOH (1 mM) at electrodes printed from Proto-pasta (Fig. S7), Prografen (Fig. S8), and Ampere (Fig. S9) after 60 s, 600 s, and 1800 s activation times in all five studied solvents (due to dissolution, different DCM activation times for electrodes printed from Proto-pasta were chosen—20 s, 40 s, and 60 s) shows that in all cases increasing activation time significantly affects electrochemical properties of the fabricated electrodes surfaces. For most cases, the evolution of clear positive and negative signals attributed to the FcMeOH oxidation/reduction, respectively, in the potential range from 0.2 to 0.6 V was recorded. With the extension of the activation time, we have observed an increase in peak current intensity in the following cases: (i) For Proto-pasta printed electrodes, this was observed for all five solvents. THF, ACN, and AC resulted in the highest Faradaic currents magnification; (ii) Prografen electrodes gave increased peak current intensities only when DCM and DCE were used. These two solvents allowed for clear differentiation of redox signals from the background; (iii) Similar to Proto-pasta, Ampere electrodes also showed a substantial increase in signal intensity when THF, ACN, and AC were used as the activation solvent. Nevertheless, DCM and DCE proved to be sufficient for activating the Ampere-electrode surface (voltammetric data available in supplementary material as Figs. S7–S9).

Along with the signal magnification, we have also noticed that for many electrodes elongated activation times lead to an increase in the peak-to-peak separation, especially for the electrodes displaying the highest signal magnification (e.g. Proto-pasta and Ampere activation in AC and ACN). This is rather a common feature of the carbon-based electrodes which may originate from (i) the saturation of the electrocatalytic sites existing at the exposed carbon-based conductive particles existing at 3DP electrode surface (lack of electrocatalytic sites at high analyte concentration); (ii) analyte adsorption; (iii) or diffusion limitation induced by the surface porosity. We assume that all three scenarios display a synergistic effect with the latter being further supported by the high roughness found with the SEM and AFM analysis and significant capacitive currents observed on some of the CVs (see Fig. S9A,D,E). Occasionally the second anodic peak shifted towards a more anodic potential value was observed (Fig S7A) which most probably indicates the adsorption process. The second peak may be attributed to the oxidation of the FcMeOH species adsorbed to the carbon sites deprived of the electrocatalytic properties.

**Figure 5:**
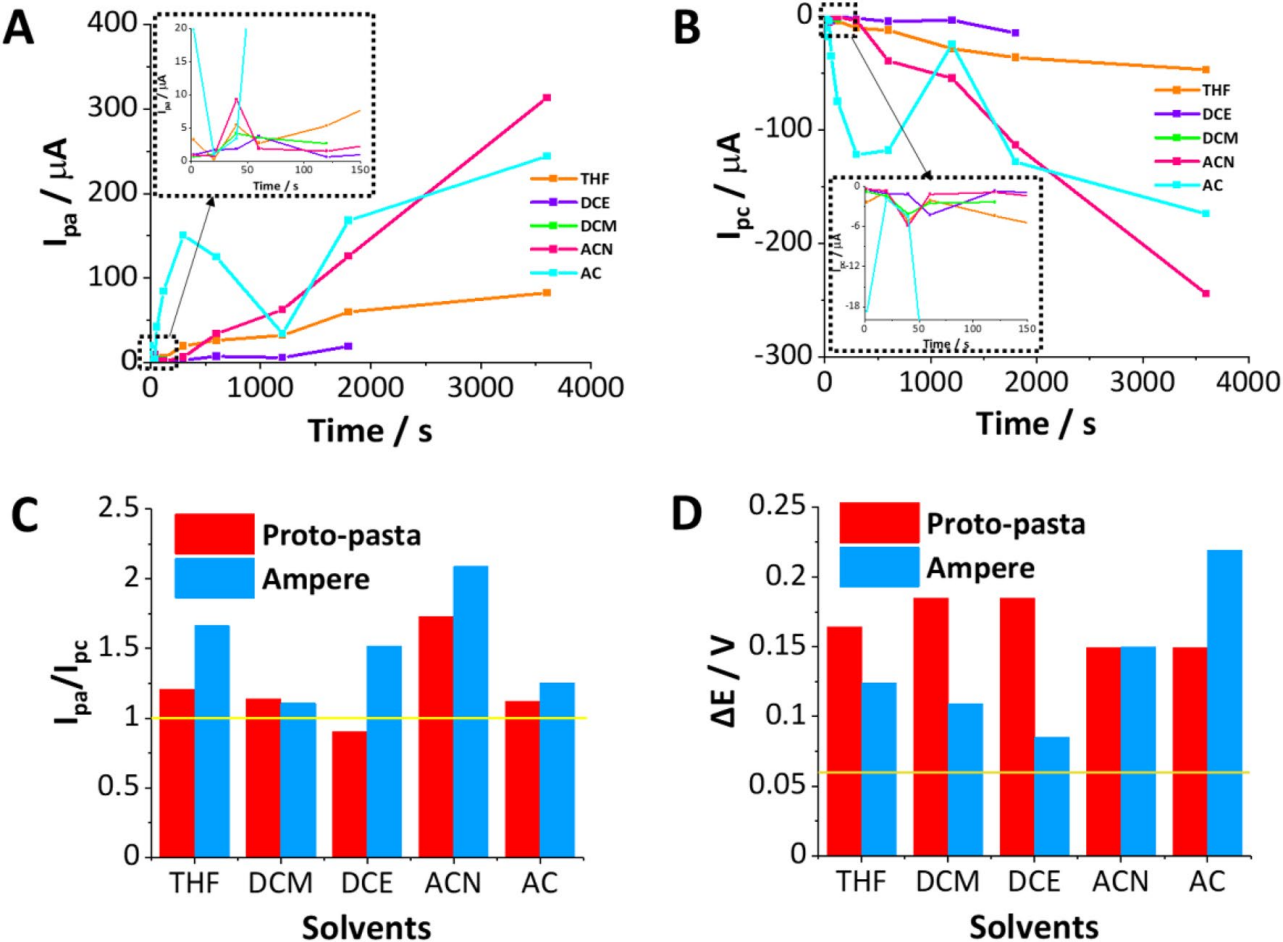
Dependence of the current intensity of the anodic (**A**) and (**B**) cathodic peaks on the solvent exposure time as recorded at the 3DP electrodes made out of Proto-pasta. Bar charts show the relationship between (**C**) the ratio of the anodic and cathodic peak currents or (**D**) peak-to-peak potential separation plotted in function of the used solvent at 120 s activation time of electrodes printed with Proto-pasta (red) and Ampere (blue). The yellow horizontal lines in panels (**C**) and (**D**) are the expected theoretical values for reversible redox reaction (I_pa_/I_pc_ = 1, ∆E = 0.059 V).

Finally, we have conducted a comprehensive analysis of all the electrochemical parameters of the studied reaction. We have focused on its reversibility, which involved assessing the ratio of the anodic to the cathodic peak current intensity (I_pa_/I_pc_) and the peak-to-peak separation (ΔE). Obtained data are plotted and depicted in Fig. 5 (data showing the results of the Proto-pasta analysis), Fig. S10 (summary of the I_pa_/I_pc_ for all three studied materials), and Fig. S11 (summary of the ΔE for all three studied materials). Figure 5A,B show the increasing intensity of the anodic and cathodic signals, respectively, plotted in function of the activation time for all five studied solvents for the 3DP electrodes fabricated using Proto-pasta. As shown, different solvents provided electrodes with different current magnification factors (as compared with the blank reading). The slope of the obtained current–time dependencies varies in a linear and increasing manner (AC activation is an exception as the fluctuation deviating from the linearity appeared in the 20–1200 s range). Interestingly, the obtained results correlate with the contact angle measurements, this is surface hydrophilicity/hydrophobicity. Based on the performed correlation, we have found that the electrodes displaying the highest anodic/cathodic currents provided the highest values of the water droplet contact angle (highest hydrophobicity). The highest values of the redox current recorded in the presence of FcMeOH at Proto-pasta-based 3DP electrodes were found when either ACN or AC activation was applied (see Fig. 5A,B). These electrodes gave the highest values of the contact angle being > 110°. In turn, the hydrophilic surface (around 60°) obtained via DCE activation gave voltammograms with good reversibility features but low current intensity. Similar correlations were also observed for electrodes made out of Ampere (results not shown). This observation may originate from two phenomena: (i) the elaborated surface roughness affected during PLA dissolution process with features that may act as the pockets for the air (high contact angle values), and at the same time significantly increased electrode surface area affecting the final current readout. (ii) Also, we can not neglect the hydrophobic interactions between the analyte and hydrophobic remainings of the oriented (conformational changes of the polymeric chains triggered by the action of organic solvents) polymeric matrix existing at the electrode surface. Although we cannot exclude the first phenomena, we believe that the hydrophobic interactions play a crucial role during the detection process as we could not observe the anodic/cathodic signals for the significantly more hydrophilic (than FcMeOH) probes such as Fe(CN)_6_^3−/4−^. The comprehensive and detailed investigation of all voltammograms (three types of materials, each activated in five solvents at multiple activation times, each electrode studied at least in triplicate) allowed the evaluation of the 3DP electrodes in terms of their electroanalytical performance as shown in Fig. 5C (I_pa_/I_pc_ for different solvents received for the electrodes printed using Protopasta or Ampere), and 5D (peak to peak separation for Proto-pasta and Ampere activated in all five solvents). Both factors are further summarized as a function of activation time in Figs. S10 and S11, respectively.

Redox probe, for which one electron takes part in the electrode reaction, should provide peak-to-peak separation not greater than 0.059 V and the anodic-to-cathodic peak intensity ratio being close to unity assuming that no additional resistance to the charge transfer reaction exists in the system. We have used these two parameters to find the activation protocol which leads to the superior 3DP electrode surface activation. Generally, activation times greater than just immersion were needed to reach the peak-to-peak separation approaching 0.059 V with the exception being Proto-pasta and Prografen activated with AC, as here the separation of the signals (along with the signal intensity) was increasing. The use of DCM activation for Proto-pasta and Ampere resulted in the ratio of the current intensity of the anodic and cathodic peaks close to the theoretical value, similar to AC activation, whereas for DCE this was only the case for Proto-pasta. However, in each of the mentioned cases, the value of peak potential separation significantly differed from 0.059 V, indicating elevated resistance of the charge transfer reaction or not efficient kinetics of the electron transfer. We assume, that whenever the I_pa_/I_pc_ deviates from the unity, the analyte adsorbs to the surface (I_pa_/I_pc_ > 1—neutral form adsorption; I_pa_/I_pc_ < 1—oxidized form adsorption). Based on the analysis of the data summary depicted in Fig. 5 and Figs. S6–S11 we can indicate a few electrode activation protocols that lead to the surface properties meeting electroanalytical demands. These are Proto-pasta electrodes activated with THF at times exceeding 20 s; Proto-pasta activated with DCE or DCM at times up to 60 s (at elongated times electrode dismantlement becomes an issue); and Ampere-based electrodes activated with DCM and DCE for times > 60 s. It seems that only the AC is recommended for the Prografen electrode treatment since other solvents either dissolved the electrode (DCM) or only affected its capacitive behavior. Also, the electroanalytical findings revealed that for the applied electrode activation procedure the measured electroanalytical signals variation (calculated based on the three repetitions for all used solvents and activation times) was always less than 5%.

#### Electroanalytical performance

**Figure 6:**
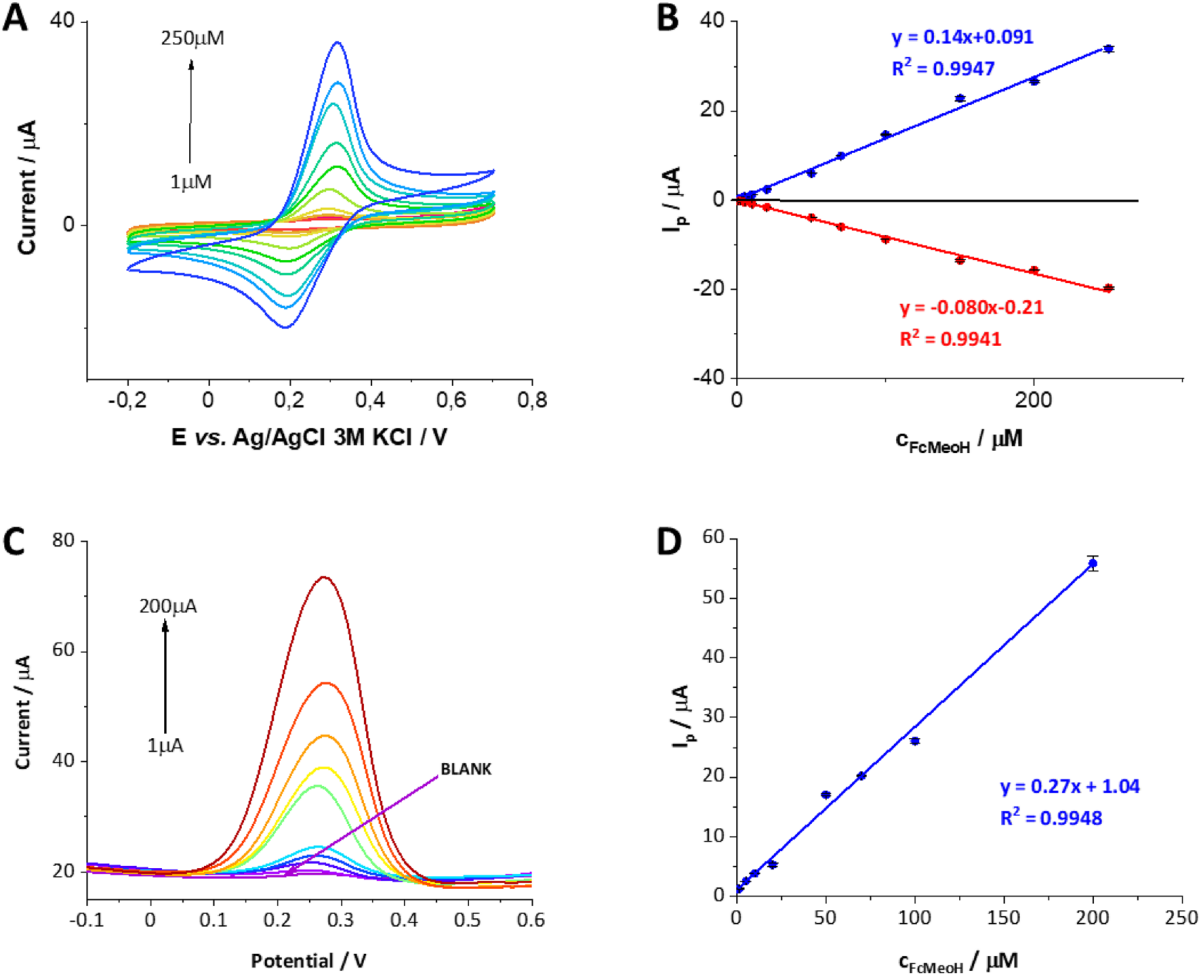
(**A**) CVs and (**C**) DPVs recorded in the presence of various concentrations of FcMeOH in 0.1 M KCl recorded using THF–activated electrode 3DP using Proto-pasta filament. (**B**) and (**D**) are the corresponding calibration plots. The error bars were constructed as confidence intervals (n = 3).

To test the electroanalytical applicability of the fabricated 3DP electrodes we have selected one procedure being a compromise between the electroanalytical output and the activation time THF activation at 600 s. The determination of FcMeOH was carried out using two techniques, i.e. CV (Fig. 6A) and DPV (Fig. 6C) which were further analyzed to provide calibration curves that are depicted in Fig. 6B,C, respectively. The analytical parameters that were determined based on the linear fit equations are presented in Table 1. The CV determination of FcMeOH showed linearity from 1 to 250 µM (Fig. 6B), and the LOD was calculated as 8.3 µM (calculated based on the anodic currents). The increasing peak-to-peak separation observed on CVs shown in Fig. S12A for higher FcMeOH concentration falling beyond the linearity range shown in Fig. 6A is most probably due to the existence of the uncompensated resistance which affects the correlation shown on the corresponding calibration curve (see Fig. S12B—second range). The use of the DPV technique for the determination of FcMeOH resulted in a slightly lower LOD calculated as 7.5 µM and an almost twofold increase in the slope of the calibration curve compared to the results obtained with the CV technique (Fig. 6D). The linearity range for this procedure was from 1 to 200 µM. This initial set of data confirms that the solvent-activated 3D printed electrodes have a high potential for electroanalytical studies. We assume, that the electrodes displaying different surface wettability which can be controlled by the time and type of the solvent applied during the activation step, will affect the sensing sensitivity and electroanalytical activity of the analytes displaying different hydrophilicity. We are currently pursuing this objective.

**Table 1:** The summary of the electroanalytical parameters obtained for FcMeOH using CV and DPV technique at the 3D printed electrode (Proto-pasta) activated with THF.

	CV	DPV
LDR (µM)	1–250	1–200
LOD (µM)	8.33	1.32
LOQ (µM)	27.8	4.00
Sensitivity (A·M^–1^)	0.14	0.27
R^2^	0.9947	0.9948

*LDR* linear dynamic range, *LOD* limit of detection, *LOQ* limit of quantification, where LOD = 3.3 SD/S and LOQ = 10 SD/S, where SD and S are the deviation of the intercept and the slope of the calibration curve, respectively (values taken from the calibration curves linear fit equation). The sensitivity of proposed analytical protocols was taken as a slope of the calibration curve.

## Conclusions

In this work, we have performed a comprehensive study to evaluate the effect of five different solvents affecting the electrochemical performance of 3D-printed electrodes fabricated using commercially available carbon-based PLA filaments (Proto-pasta, Prografen, and Ampere). The solvents that were chosen were acetone, acetonitrile, dichloromethane, dichloroethane, and tetrahydrofuran. We have found that all can dissolve PLA to different extents (as expected chlorinated solvents displayed the highest solvent power towards PLA). The electrodes were studied with cyclic voltammetry, atomic force microspecies, scanning electron microscopy, and contact angle analysis. Obtained data suggest the correlation between surface hydrophobicity/hydrophilicity (contact angle measurements), the surface appearance inferred from the imaging study, and finally electroanalytical output. Electrode surface displaying the highest hydrophilicity were also the one providing high surface roughness and good electroanalytical output. Dichloroethane, dichloromethane and tetrahydrofuran were found to be the best solvent for the electrode surface activation. Finally, we have chosen one activation procedure providing satisfactory electrochemical properties and used the 3D printed electrode to assess analytical characteristics based on the model redox probe detection (ferrocene methanol). Cyclic voltammetry and differential pulse voltammetry were used in this respect.

### Supplementary Information

Supplementary Figures.

## Data Availability

The datasets generated and/or analysed during the current study are available in the Zenodo repository, https://zenodo.org/record/8027817.
