# Supplementary material for: Solvent-activated 3D-printed electrodes and their electroanalytical potential
Source: Sci Rep. 2023 Dec 20;13:22797. doi: 10.1038/s41598-023-49599-9 (PMC10739953; doi:10.1038/s41598-023-49599-9)
Supplement: Supplementary file 1 — Supplementary Figures. [file 41598_2023_49599_MOESM1_ESM.docx]

**Solvent-activated 3D-printed electrodes and their electroanalytical potential**

Karolina Kwaczyński^1,*^, Olga Szymaniec^1^, Diana M. Bobrowska^2^, Łukasz Półtorak^1,*^

*^1^University of Lodz, Faculty of Chemistry, Department of Inorganic and Analytical Chemistry, Tamka 12, 91-403 Lodz, Poland*

*^2^ University of Bialystok, Faculty of Chemistry, Ciolkowskiego 1K, 15-245, Bialystok, Poland*

*First corresponding author: karolina.kwaczynski@chemia.uni.lodz.pl

*Second corresponding author: lukasz.poltorak@chemia.uni.lodz.pl

**Table of content:**

- **Page 03 - Figure S1.** Real photo of 3D printed electrodes made out of (A) Proto-pasta, (B) Prografen, and (C) Ampere.
- **Page 04** - **Figure S2.** Real photos of printouts made using (A) bare PLA, (B) Proto-paste, (C) Prografen, and (D) Ampere that were exposed to THF, DCM, DCE, AC, and ACN solvents for a specified time (immersion, 20s, 40s, 60s, 120s, 600s, 1200s, 1800s, and 3600s).
- **Page 05** - **Figure S3.** SEM images of 3D-printed electrodes from **(A)** Proto-pasta, **(B)** Prografen, **(C)** Ampere after **(I)** THF, **(II)** DCE, **(III)** DCM, **(IV)** AC, **(V)** ACN-activation for 120s. Magnification: 5 000x.
- **Page 06** - **Figure S4.** AFM images and corresponding surface roughness height profiles of 3DP electrodes made out of Proto-pasta and Ampere (see labels) after DCE-activation for 120s.
- **Page 07** – **Figure S5.** Cyclic voltammograms (CVs) recorded in the aqueous solution of 1 mM FcMeOH in 0.1 M KCl before (non-activated surfaces – black, dashed lines) and after 3D printed electrodes activation with an indicated solvent (solid lines: orange – THF, violet – DCE, pink – ACN). The activation time was set to 300s. The filament type is indicated in the left panel of the figure. The scan rate was 100 mV·s^-1^. The anodic scan was set as the forward polarization. The data set showing the uniform y-axes further indicating the changes of the electroactive surface.
- **Page 08** – **Figure S6.** Cyclic voltammograms (CVs) recorded in 1 mM FcMeOH in 0.1 M KCl solution at nonactivated (black, solid line; ) and activated with a suitable solvent (light green – DCM, cyan – AC) for 300s 3DP electrodes. DCM-activated Proto-Pasta electrode is an exception as here the CVs correspond to 120s activation time. The name of the used filament is indicated in the left panel.
- **Page 09** – **Figure S7.** Cyclic voltammograms (CV) recorded in 1 mM FcMeOH in 0.1 M KCl solution at nonactivated (black, dashed line) and activated with (A) THF, (B) DCM, (C) DCE, (D) ACN, and (E) AC for 60s, 600s, and 1800s (except for the DCM-activated electrode, activation times were 20s, 40s, and 60s) 3DP electrodes. Proto-paste was used as the carbon-based filament for the electrode fabrication.
- **Page 010** – **Figure S8.** Cyclic voltammograms (CV) recorded in 1 mM FcMeOH in 0.1 M KCl solution at nonactivated (black, dashed line) and activated Prografen 3DP electrodes. Activation was performed with (A) THF, (B) DCM, (C) DCE, (D) ACN, and (E) AC for 60s, 600s, and 1800s.
- **Page 11** - **Figure S9.** Cyclic voltammograms (CV) recorded in 1 mM FcMeOH in 0.1 M KCl solution at nonactivated (black, dashed line) and Ampere-based 3DP electrodes activated with (A) THF, (B) DCM, (C) DCE, (D) ACN, and (E) AC for 60s, 600s, and 1800s.
- **Page 12** - **Figure 10.** The dependency between the ratio of anodic to cathodic peak current plotted in function of the activation time (2s, 20s, 60s, 300s) for all studied solvents (THF, DCM, DCE, ACN, AC). Figures are labeled as follows: (A) Proto-paste, (B ) Prografen, (C) Ampere. The yellow horizontal corresponds to I_pa_/I_pc_ = 1.
- **Page 13** - **Figure S11.** The dependency between the peak potential separation plotted in function of the activation time (2s, 20s, 60s, 300s) for all studied solvents (THF, DCM, DCE, ACN, AC). Figure labels correspond to (A) Proto-paste, (B ) Prografen, (C) Ampere. The yellow horizontal line corresponds to ΔE = 0.059 V.
- **Page 14** – **Figure S12.** (A) CVs recorded in the presence of various concentrations of FcMeOH in 0.1 M KCl recorded using THF–activated electrode 3DP using Proto–Pasta filament. (B) The corresponding calibration plot. The error bars were constructed as confidence intervals (n = 3).

**Raw data can be found through the DOI**: 10.5281/zenodo.8027817


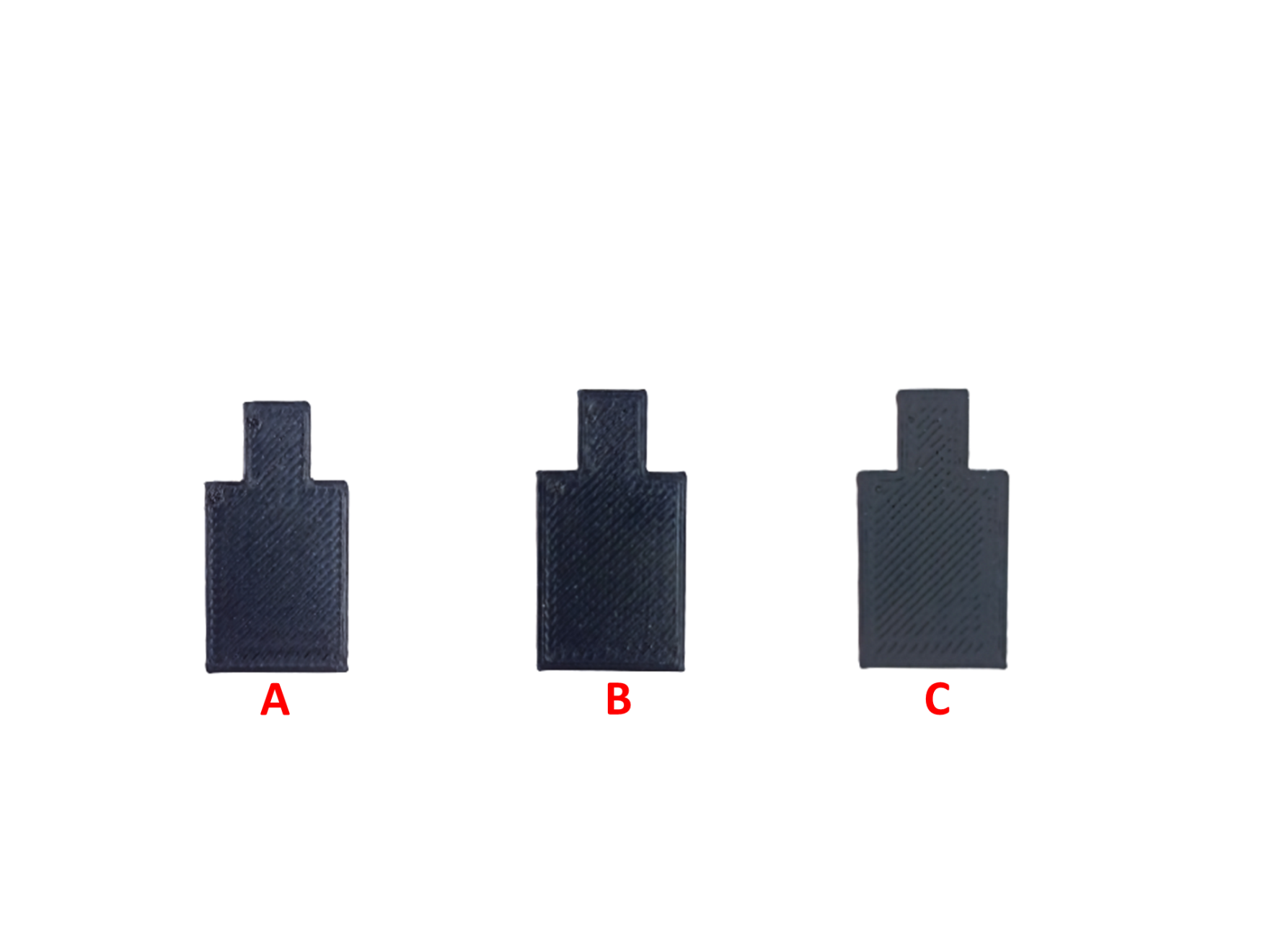


**Figure S1.** Real photo of 3D printed electrodes made out of (A) Proto-pasta, (B) Prografen, and (C) Ampere.


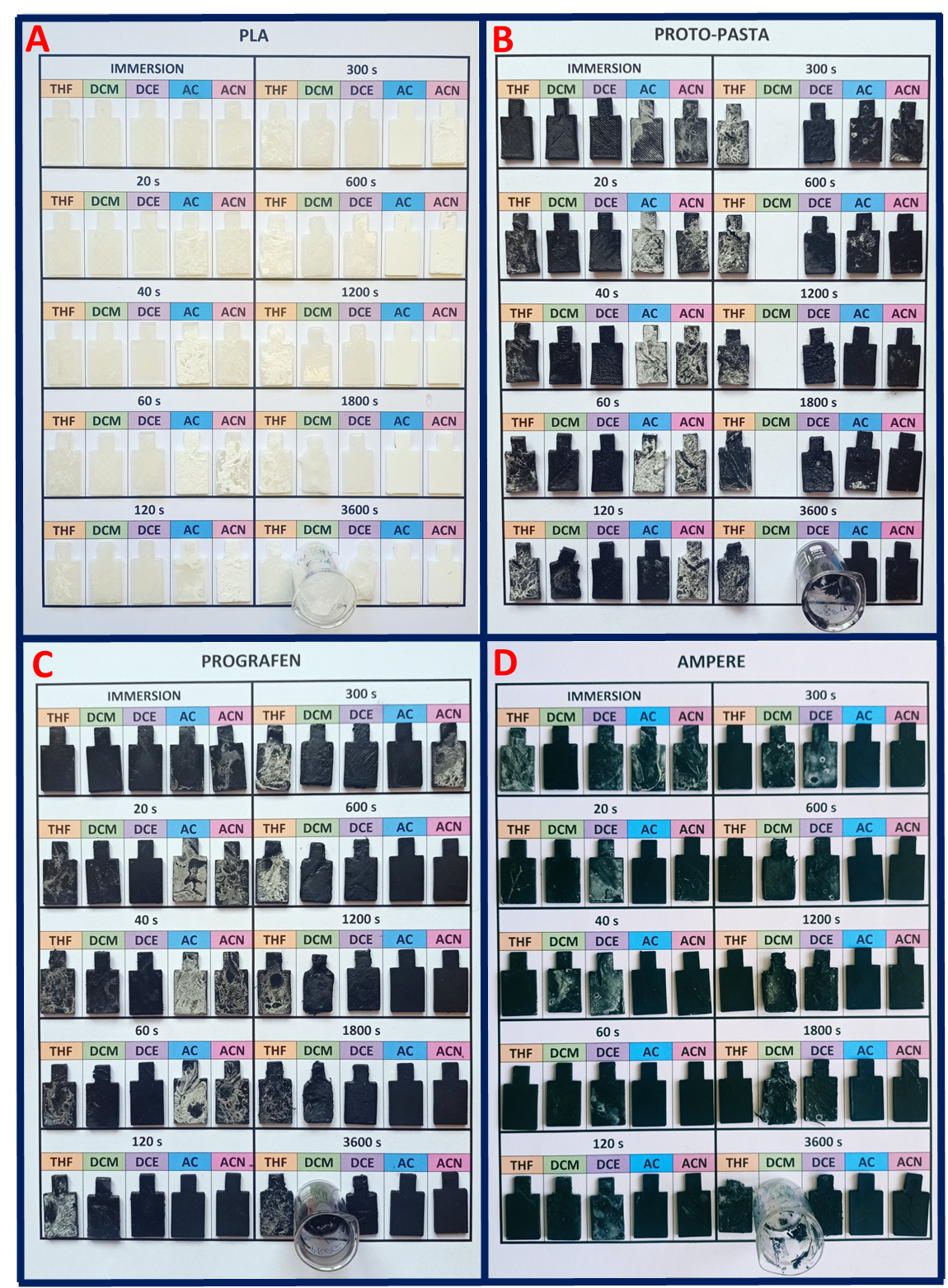


**Figure S2.** Real photos of print outs made using (A) bare PLA, (B) Proto-paste, (C) Prografen, and (D) Ampere that were exposed to THF, DCM, DCE, AC, and ACN solvents for a specified time (immersion, 20s, 40s, 60s, 120s, 600s, 1200s, 1800s, and 3600s).


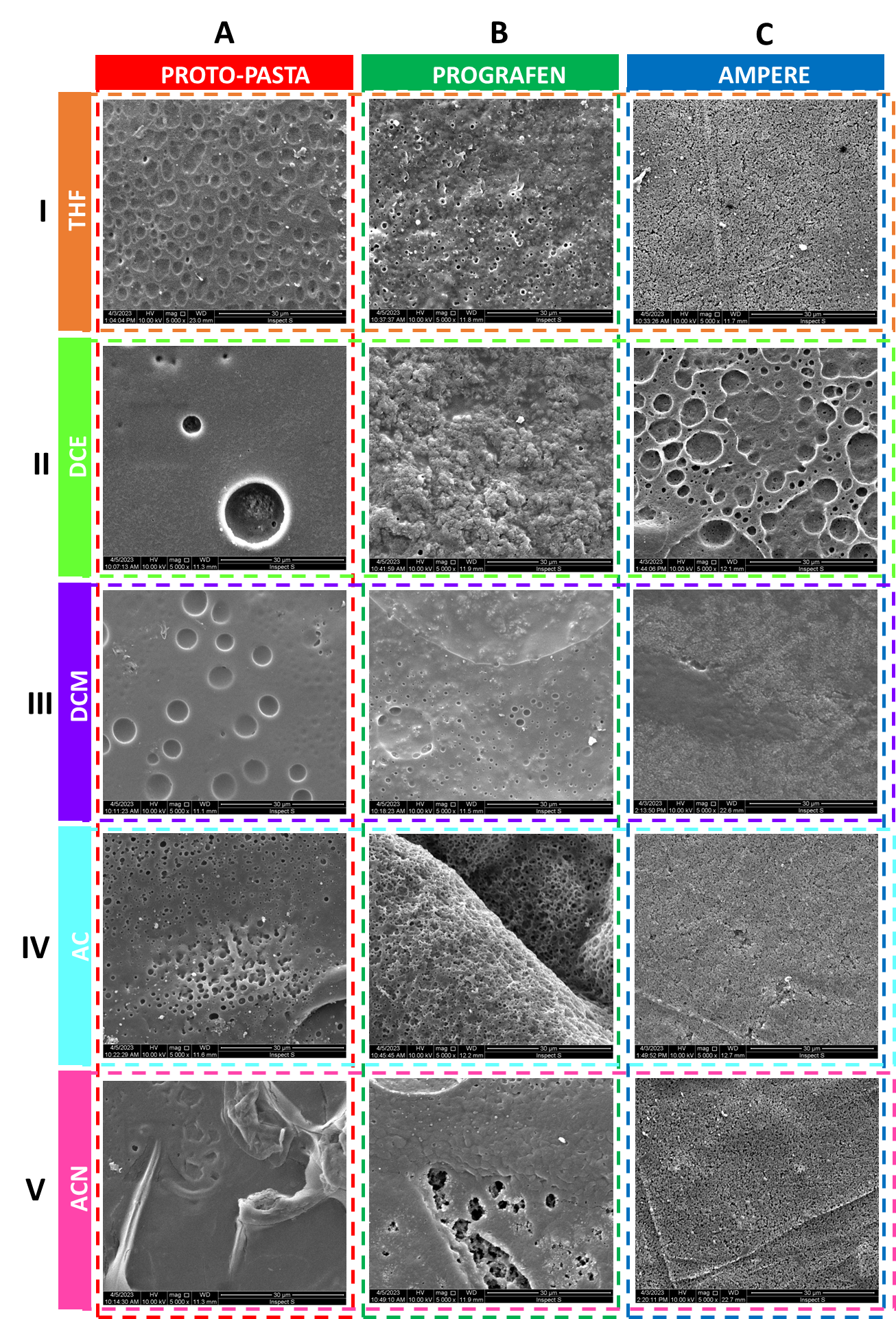


**Figure S3.** SEM images of 3D-printed electrodes from **(A)** Proto-pasta, **(B)** Prografen, **(C)** Ampere after **(I)** THF, **(II)** DCE, **(III)** DCM, **(IV)** AC, **(V)** ACN-activation for 120s. Magnification: 5 000x.


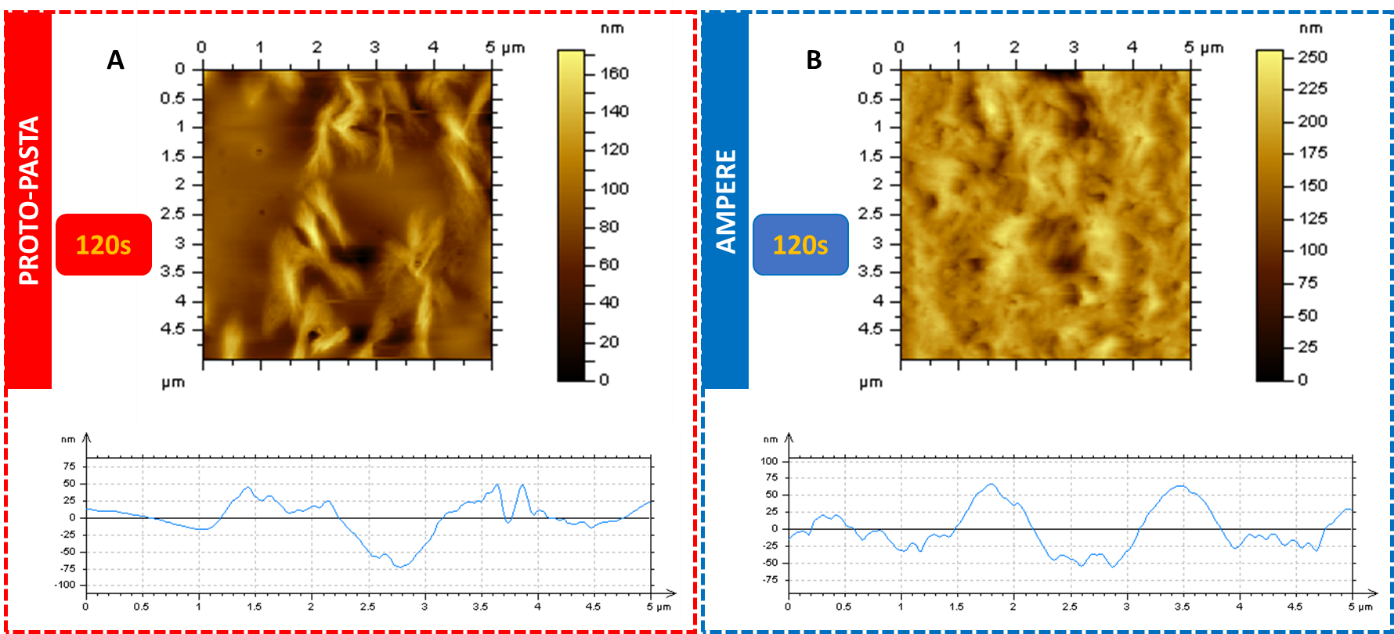


**Figure S4.** AFM images and corresponding surface roughness height profiles of 3DP electrodes made out of (A) Proto-pasta and (B)Ampere after DCE-activation for 120s.


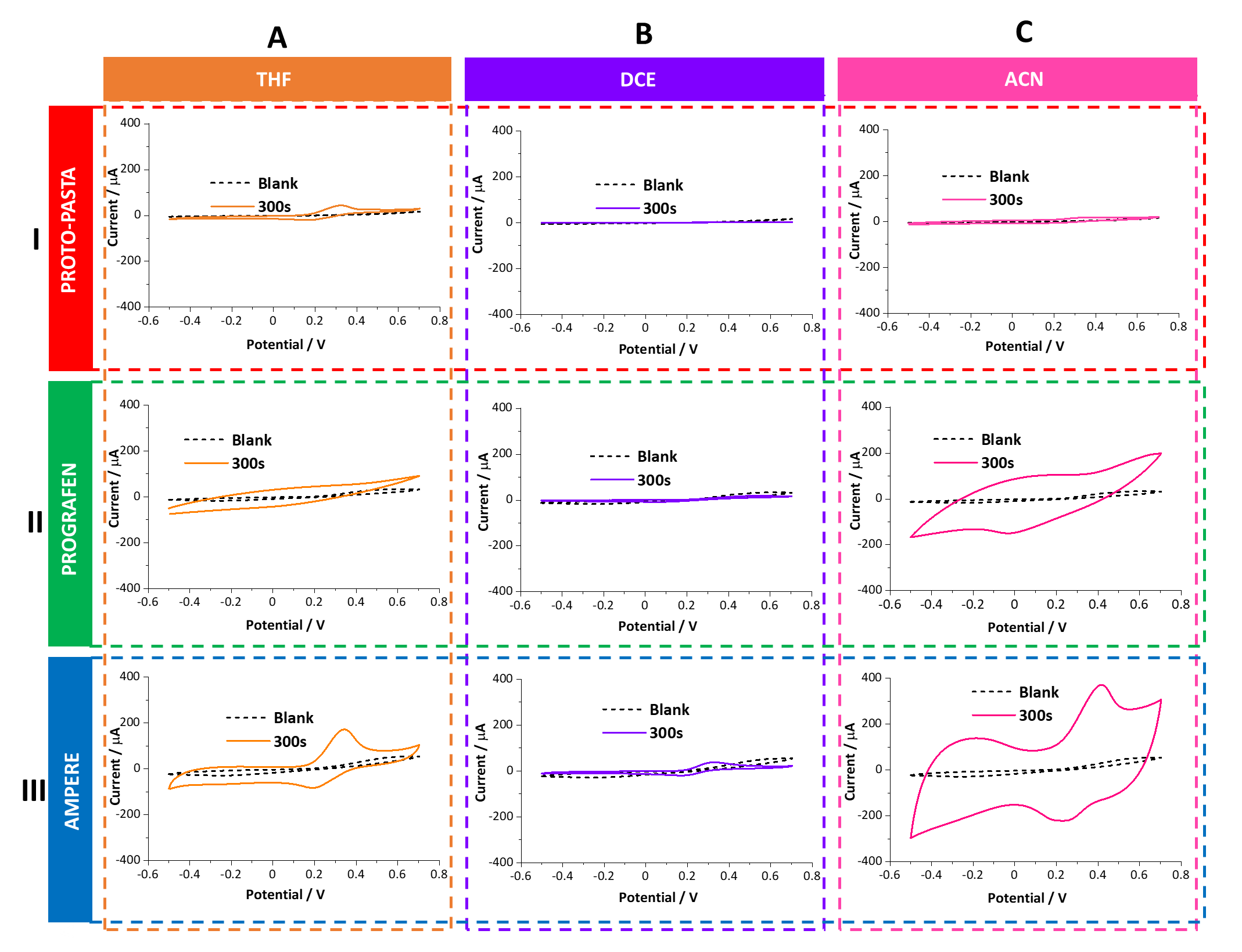


**Figure S5.** Cyclic voltammograms (CVs) recorded in the aqueous solution of 1 mM FcMeOH in 0.1 M KCl before (non-activated surfaces – black, dashed lines) and after 3D printed electrodes activation with an indicated solvent (solid lines: orange – THF, violet – DCE, pink – ACN). The activation time was set to 300s. The filament type is indicated in the left panel of the figure. The scan rate was 100 mV·s^-1^. The anodic scan was set as the forward polarization. The data set showing the uniform y-axes further indicating the changes of the electroactive surface.


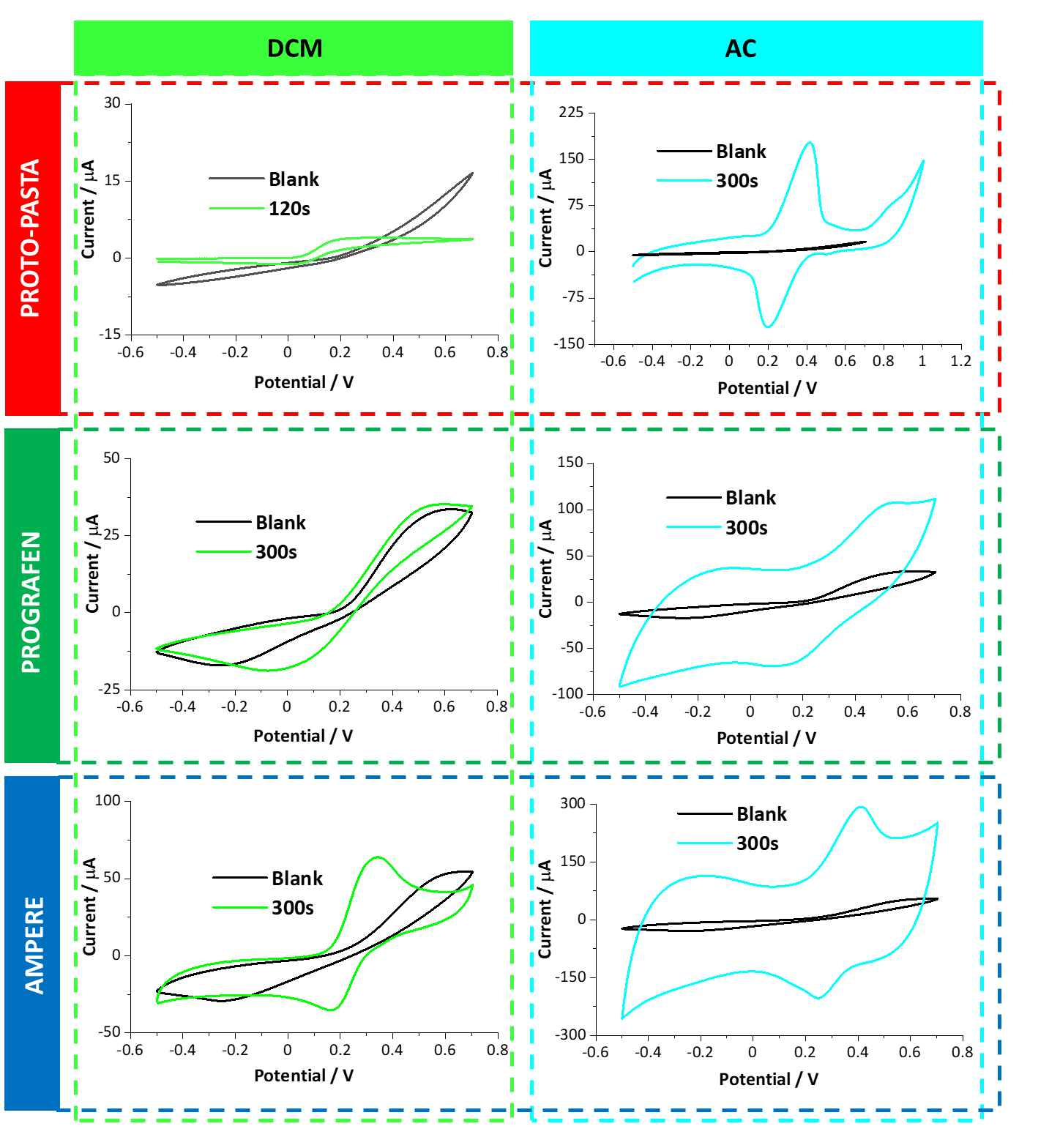


**Figure S6.** Cyclic voltammograms (CVs) recorded in 1 mM FcMeOH in 0.1 M KCl solution at nonactivated (black, solid line; ) and activated with a suitable solvent (light green – DCM, cyan – AC) for 300s 3DP electrodes. DCM-activated Proto-Pasta electrode is an exception as here the CVs correspond to 120s activation time. The name of the used filament is indicated in the left panel.


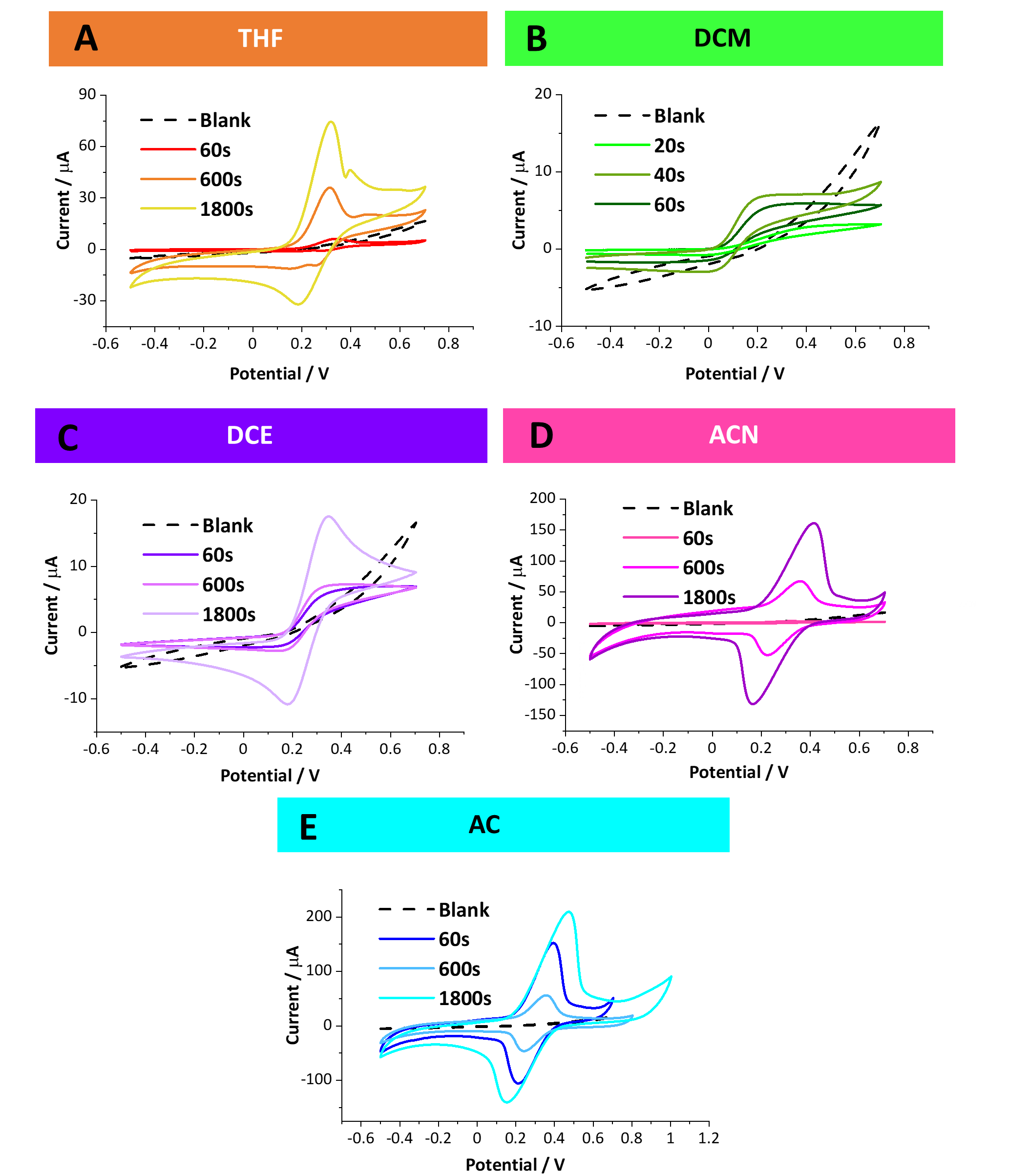


**Figure S7.** Cyclic voltammograms (CV) recorded in 1 mM FcMeOH in 0.1 M KCl solution at nonactivated (black, dashed line) and activated with (A) THF, (B) DCM, (C) DCE, (D) ACN, and (E) AC for 60s, 600s, and 1800s (except for the DCM-activated electrode, activation times were 20s, 40s, and 60s) 3DP electrodes. Proto-paste was used as the carbon based filament for the electrodes fabrication.

**
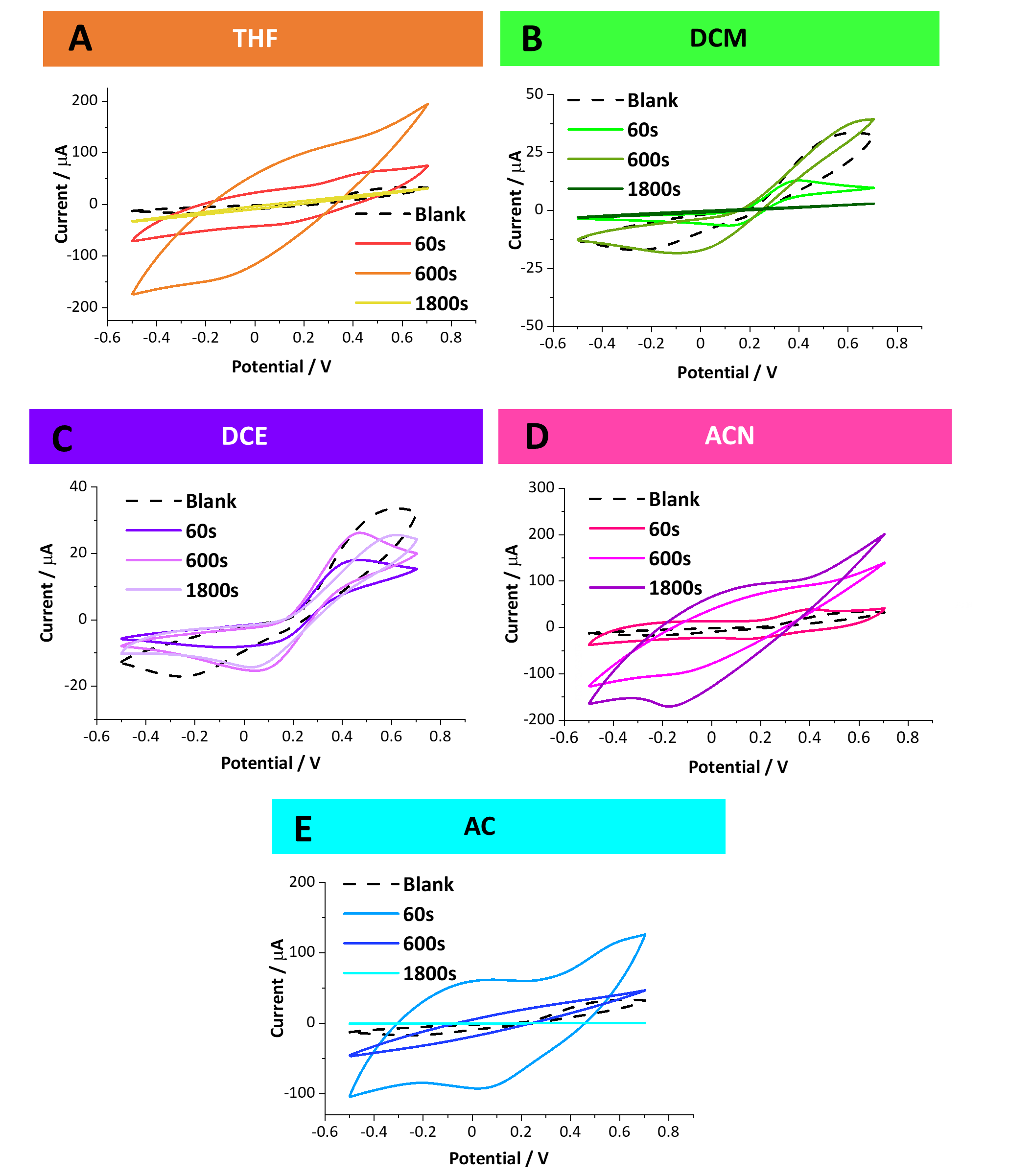
**

**Figure S8.** Cyclic voltammograms (CV) recorded in 1 mM FcMeOH in 0.1 M KCl solution at nonactivated (black, dashed line) and activated Prografen 3DP electrodes. Activation was performed with (A) THF, (B) DCM, (C) DCE, (D) ACN, and (E) AC for 60s, 600s, and 1800s.


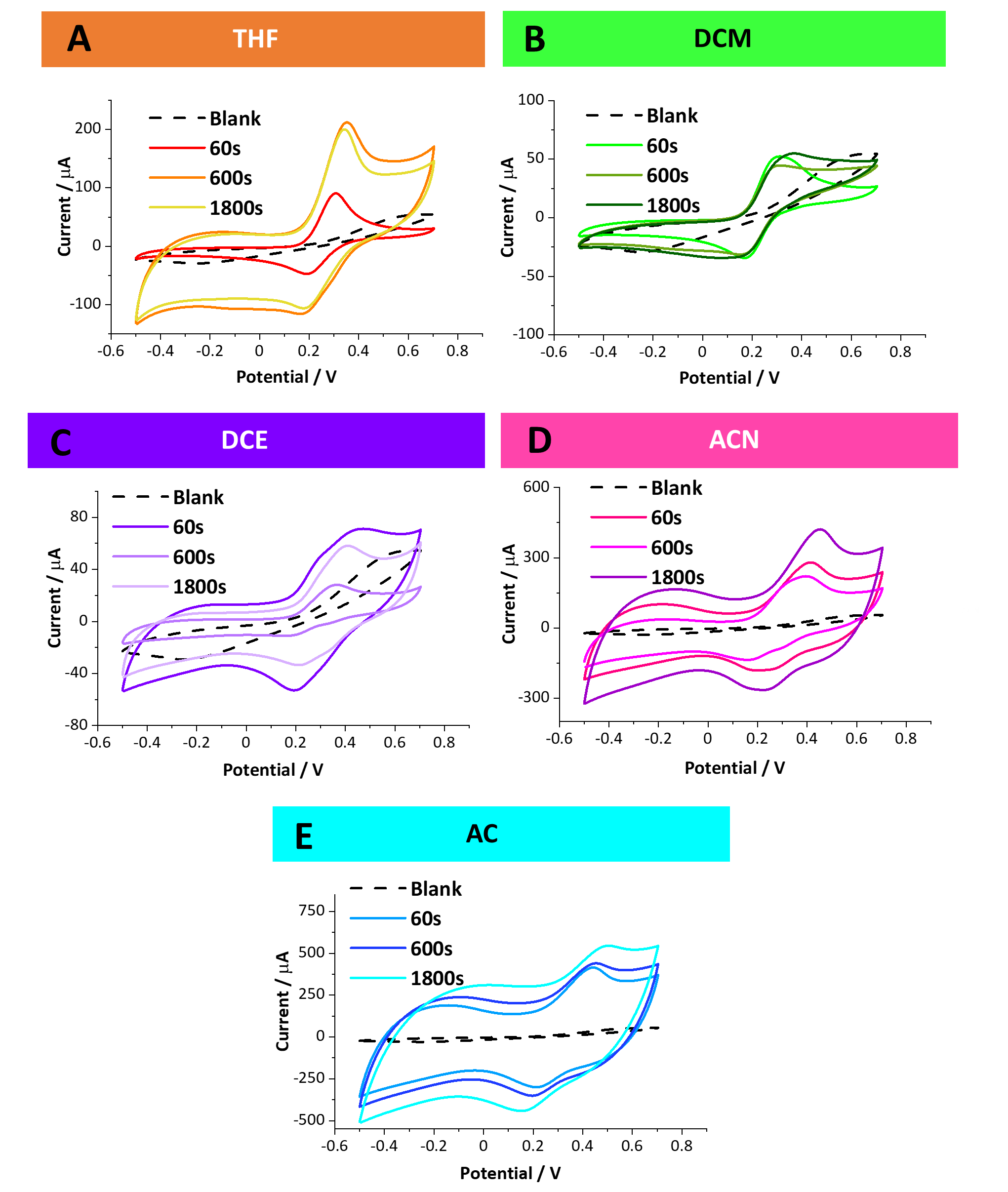


**Figure S9.** Cyclic voltammograms (CV) recorded in 1 mM FcMeOH in 0.1 M KCl solution at nonactivated (black, dashed line) and Ampere based 3DP electrodes activated with (A) THF, (B) DCM, (C) DCE, (D) ACN, and (E) AC for 60s, 600s, and 1800s.


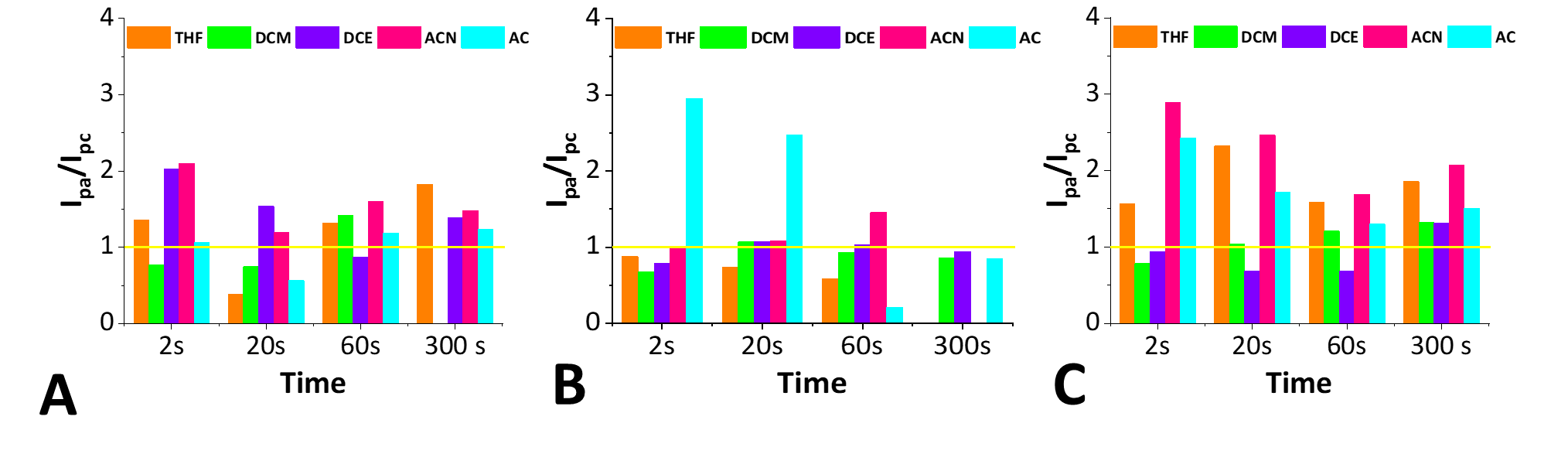


**Figure S10.** The dependency between the ratio of anodic to cathodic peak current plotted in function of the activation time (2s, 20s, 60s, 300s) for all studied solvents (THF, DCM, DCE, ACN, AC). Figures are labeled as follows: (A) Proto-paste, (B ) Prografen, (C) Ampere. The yellow horizontal corresponds to I_pa_/I_pc_ = 1.


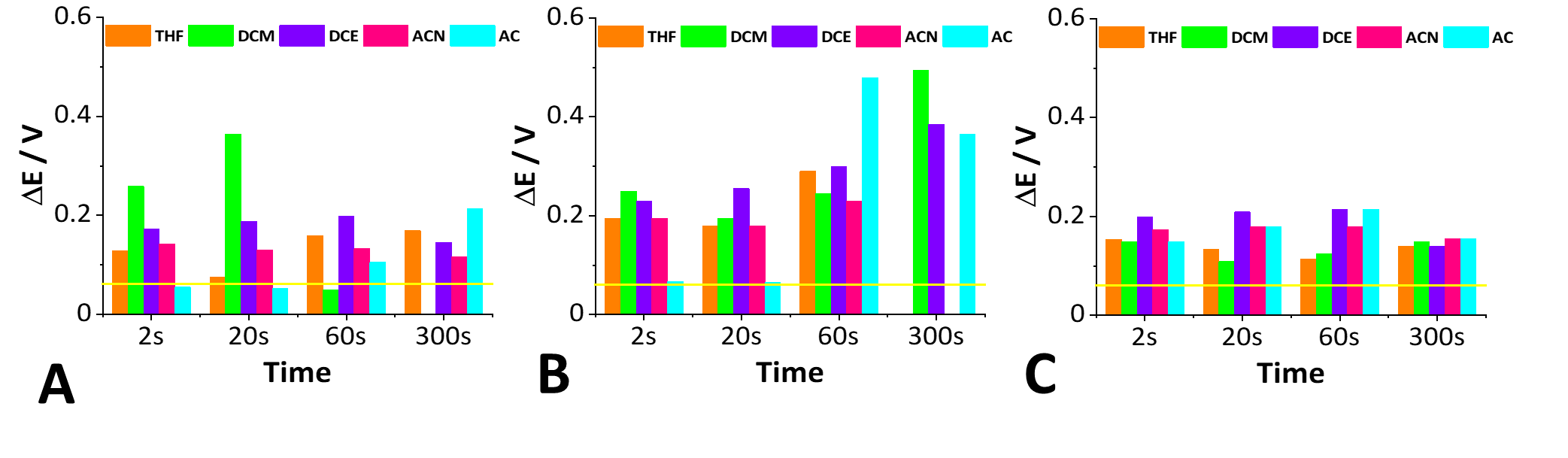


**Figure S11.** The dependency between the peak potential separation plotted in function of the activation time (2s, 20s, 60s, 300s) for all studied solvents (THF, DCM, DCE, ACN, AC). Figure labels correspond to (A) Proto-paste, (B ) Prografen, (C) Ampere. The yellow horizontal line correspond to ΔE = 0.059 V.

**
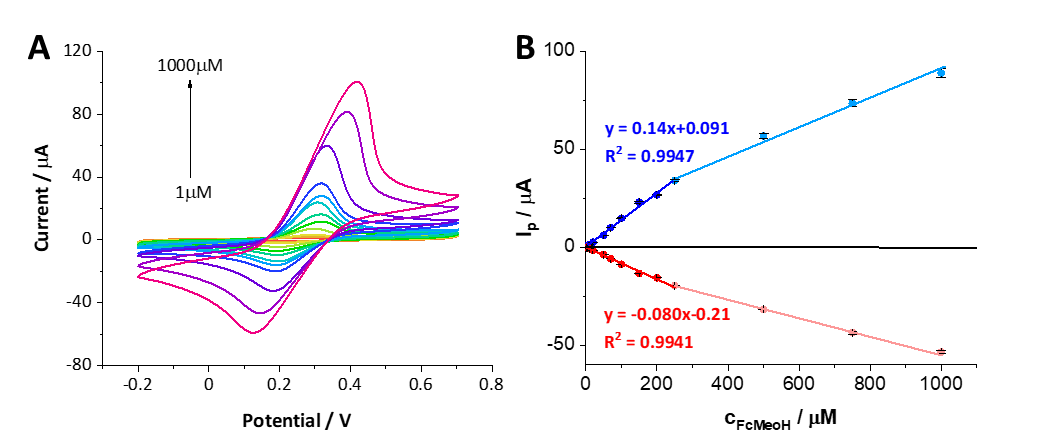
**

**Figure S12.** (A) CVs recorded in the presence of various concentrations of FcMeOH in 0.1 M KCl recorded using THF–activated electrode 3DP using Proto–Pasta filament. (B) The corresponding calibration plot. The error bars were constructed as confidence intervals (n = 3).
